# *Ehrlichia* Wnt SLiM ligand mimic deactivates the Hippo pathway to engage the anti-apoptotic Yap-GLUT1-BCL-xL axis

**DOI:** 10.1128/iai.00085-23

**Published:** 2023-08-02

**Authors:** Caitlan D. Byerly, LaNisha L. Patterson, Nicholas A. Pittner, Regina N. Solomon, Jignesh G. Patel, Madison R. Rogan, Jere W. McBride

**Affiliations:** 1 Department of Pathology, University of Texas Medical Branch, Galveston, Texas, USA; 2 Department of Microbiology and Immunology, University of Texas Medical Branch, Galveston, Texas, USA; 3 Center for Biodefense and Emerging Infectious Diseases, University of Texas Medical Branch, Galveston, Texas, USA; 4 Sealy Institute for Vaccine Sciences, University of Texas Medical Branch, Galveston, Texas, USA; 5 Institute for Human Infections and Immunity, University of Texas Medical Branch, Galveston, Texas, USA; Washington State University, Pullman, Washington, USA

**Keywords:** *Ehrlichia*, Hippo signaling, effector, ligand, molecular mimicry, short linear motif, tandem repeat protein

## Abstract

*Ehrlichia chaffeensis* TRP120 effector has evolved short linear motif (SLiM) ligand mimicry to repurpose multiple evolutionarily conserved cellular signaling pathways, including Wnt, Notch, and Hedgehog. In this investigation, we demonstrate that *E. chaffeensis* and recombinant TRP120 deactivate Hippo signaling, resulting in the activation of Hippo transcription coactivator Yes-associated protein (Yap). Moreover, a homologous 6 amino acid (QDVASH) SLiM shared by TRP120 and Wnt3a/5a ligands phenocopied Yap and β-catenin activation induced by *E. chaffeensis,* rTRP120, and Wnt5a. Similar Hippo gene expression profiles were also stimulated by *E. chaffeensis*, rTRP120, SLiM, and Wnt5a. Single siRNA knockdown of Hippo transcription co-activator/factors, Yap, and transcriptional enhanced associate domain (TEAD) significantly decreased *E. chaffeensis* infection. Yap activation was abolished in THP-1 Wnt Frizzled-5 (Fzd5) receptor knockout cells (KO), demonstrating Fzd5 receptor dependence. In addition, the TRP120-Wnt-SLiM antibody blocked Hippo deactivation (Yap activation). Expression of anti-apoptotic Hippo target gene *SLC2A1* (encodes glucose transporter 1; GLUT1) was upregulated by *E. chaffeensis* and corresponded to increased levels of GLUT1. Conversely, siRNA knockdown of *SLC2A1* significantly inhibited infection. Higher GLUT1 levels correlated with increased B cell lymphoma-extra large (BCL-xL) and decreased BCL2-associated X, apoptosis regulator (Bax) levels. Moreover, blocking Yap activation with the inhibitor Verteporfin induced apoptosis that corresponded to significant reductions in GLUT1 and BCL-xL levels and activation of Bax and Caspase-3 and -9. This study identifies a novel shared Wnt/Hippo SLiM ligand mimic and demonstrates that *E. chaffeensis* deactivates the Hippo pathway to engage the anti-apoptotic Yap-GLUT1-BCL-xL axis.

## INTRODUCTION

*Ehrlichia chaffeensis* is a Gram-negative, obligatory intracellular bacterium and the etiologic agent of the life-threatening tick-borne disease, human monocytic ehrlichiosis (HME) (1, 2). It preferentially infects mononuclear phagocytes, where it replicates within cytosolic, membrane-bound vacuoles and escapes host defenses through mechanisms executed by tandem repeat protein (TRP) effectors secreted by the type 1 secretion system (T1SS) (1). In the past decade, *E. chaffeensis* 120 kDa tandem repeat protein (TRP120) has emerged as a model moonlighting effector that functions as a nucleomodulin, ubiquitin ligase, and ligand mimic to reprogram the mononuclear phagocyte and escape host innate immune defenses (3 - 5). TRP120 utilizes ligand mimicry to interact with various receptors to reprogram host cell signaling pathways conserved among eukaryotes, including Wnt, Notch, and Hedgehog, via novel tandem repeat short linear motifs (SLiMs) within the TRP domain (6 - 10). Ligand mimicry enables pathogens to impersonate the function of eukaryote ligands for infection (11).

We previously demonstrated that *E. chaffeensis* activates canonical Wnt signaling by direct interaction between a TRP120-Wnt-SLiM ligand mimic and the host cell Wnt Frizzled 5 (Fzd5) receptor (7). Interactions between canonical Wnt ligands and the Fzd5 receptor are known to stimulate Wnt transcriptional factor β-catenin but can also result in the deactivation of Hippo signaling, which coincides with the activation of transcription regulator Yes-associated protein (Yap) through Wnt-Hippo/Fzd receptor crosstalk (12). SLiMs are short (3–11 amino acids) linear sequences typically found within intrinsically disordered protein domains that are responsible for mediating various cellular processes (13, 14). Interestingly, there are 23 predicted SLiMs in 14 proteins involved in Wnt signal transduction, including Axin, Dishevelled, and β-catenin (15). However, Wnt ligand SLiMs that mimic endogenous eukaryotic ligands, leading to pathway activation, have only recently been identified in *Ehrlichia* (7).

The Hippo pathway, discovered in *Drosophila* in 2003, is evolutionarily conserved in metazoans and universally recognized as a key regulator in embryogenesis, organ size, tissue homeostasis, cell proliferation, apoptosis, and tumorigenesis (16 - 20). Typically, when the Hippo pathway is active, the downstream transcriptional co-activator Yap is phosphorylated and deactivated, preventing nuclear translocation and activation of Hippo gene targets. Recently, investigations have linked pathway crosstalk between the Wnt and Hippo signaling pathways to control cell fate, demonstrating that Wnt5a and Wnt3a ligands bind the Fzd5 receptor to deactivate Hippo signaling and activate Yap (12, 20, 21). Additional crosstalk occurs between Yap and Wnt transcriptional regulator, β-catenin. When Hippo is active, phosphorylated Yap remains within the cytoplasm, where it sequesters β-catenin, leading to the degradation of β-catenin and inhibition of Wnt signaling (20). Thus, Hippo deactivation through interactions between Wnt5a/Wnt3a and Fzd5 receptor must occur to support Wnt signaling. Notably, *E. chaffeensis* is a known β-catenin activator and utilizes a TRP120-Wnt-SLiM to activate β-catenin for Wnt gene regulation (7).

Regulation of apoptosis as a survival strategy is well documented during *E. chaffeensis* infection (22). Mitochondria are the primary regulators of apoptosis by both intrinsic and extrinsic pathways; thus, inhibition of mitochondrial outer membrane permeabilization (MOMP) is required to prevent apoptosis. It is known that *E. chaffeensis* utilizes the eukaryotic translation termination factor-1 (Etf-1) effector to stabilize mitochondria by regulating mitochondrial matrix protein manganese superoxide dismutase (MnSOD) to induce antioxidative protection and inhibit apoptosis (22). Further, *E. chaffeensis* utilizes a TRP120 Hedgehog SLiM ligand mimic to activate Hedgehog signaling, which prevents intrinsic apoptosis by maintaining B-cell lymphoma 2 (BCL2) levels and mitochondrial stability (10). However, there are likely other mechanisms *E. chaffeensis* engages to stabilize mitochondria, such as modulating the anti-apoptotic BCL2 family of proteins, including B-cell lymphoma-extra large (BCL-xL), which is regulated by the Hippo pathway (23).

The Hippo pathway regulates various innate and metabolic responses, including glycolysis and apoptosis (24 - 26). When Hippo signaling is deactivated, activated Yap binds the transcriptional enhanced associate domain (TEAD) and induces *SLC2A1* [encodes glucose transporter 1 (GLUT1)] upregulation, thereby promoting glycolysis and cell growth and apoptosis inhibition (23, 26 - 28). GLUT1 is a highly conserved glucose transporter that regulates glucose metabolism and prevents apoptosis by regulating the BCL-2 family of proteins. Specifically, GLUT1 amplifies anti-apoptotic BCL-xL levels and inhibits Bax to prevent caspase activation, resulting in an anti-apoptotic environment (23, 26, 29 - 32). In contrast, GLUT1 deficiency induces expression of pro-apoptotic proteins Bax, BCL2 antagonist/killer 1 (Bak), Bcl-2-like protein 11 (Bim), and BH3 interacting-domain death agonist (Bid) and inhibits expression of anti-apoptotic proteins myeloid cell leukemia-1 (MCL-1) and BCL-xL (33).

The Hippo pathway is well known for its role in cancer but has recently been implicated in viral infections, including Hepatitis B virus (HBV), Hepatitis C virus (HCV), Human papillomavirus (HPV), Epstein–Barr virus (EBV), and Kaposi Sarcoma-associated herpesvirus (KSHV) (34). However, there are only a few reports of Hippo exploitation by parasites, fungi, and bacteria (35 - 37). Yap activation by viruses has been reported, but the precise mechanism whereby deactivation of Hippo signaling occurs to activate Yap remains unclear (34). Studies have shown that Yap activation during HBV infection triggers hepatocarcinogenesis and pathogenesis of the liver and may cause HBV-induced hepatocellular carcinoma. Additionally, HBV infection in Alb-preΔS2 transgenic mice increases the expression of Hippo target genes *BIRC5*, *ANKRD1*, *CTGF*, and *CYR61* (34, 38). HPV E6 major oncoprotein inhibits active Yap degradation, and Yap knockdown impairs E6-mediated cell proliferation, indicating that Yap activation plays a role in the proliferation of cervical cancer cells (39).

Although the Hippo pathway is targeted by multiple pathogens, the pathogen–host interactions and mechanisms involved in Hippo pathway exploitation have not been defined. We have previously identified an *Ehrlichia* Wnt SLiM ligand mimic that activates Wnt signaling. Therefore, since Hippo signaling is initiated through Wnt Fzd receptors, we considered that Hippo signaling may be regulated through the same ligand–receptor complex during infection (40). This investigation reveals a molecular mechanism whereby *E. chaffeensis* utilizes a eukaryotic Wnt SLiM ligand motif interaction with the Fzd5 receptor to deactivate Hippo signaling, thereby activating the Yap-GLUT1-BCL-xL axis to promote an anti-apoptotic cellular environment.

## RESULTS

### *E. chaffeensis* activates Yap and Hippo gene expression

**Fig 1:**
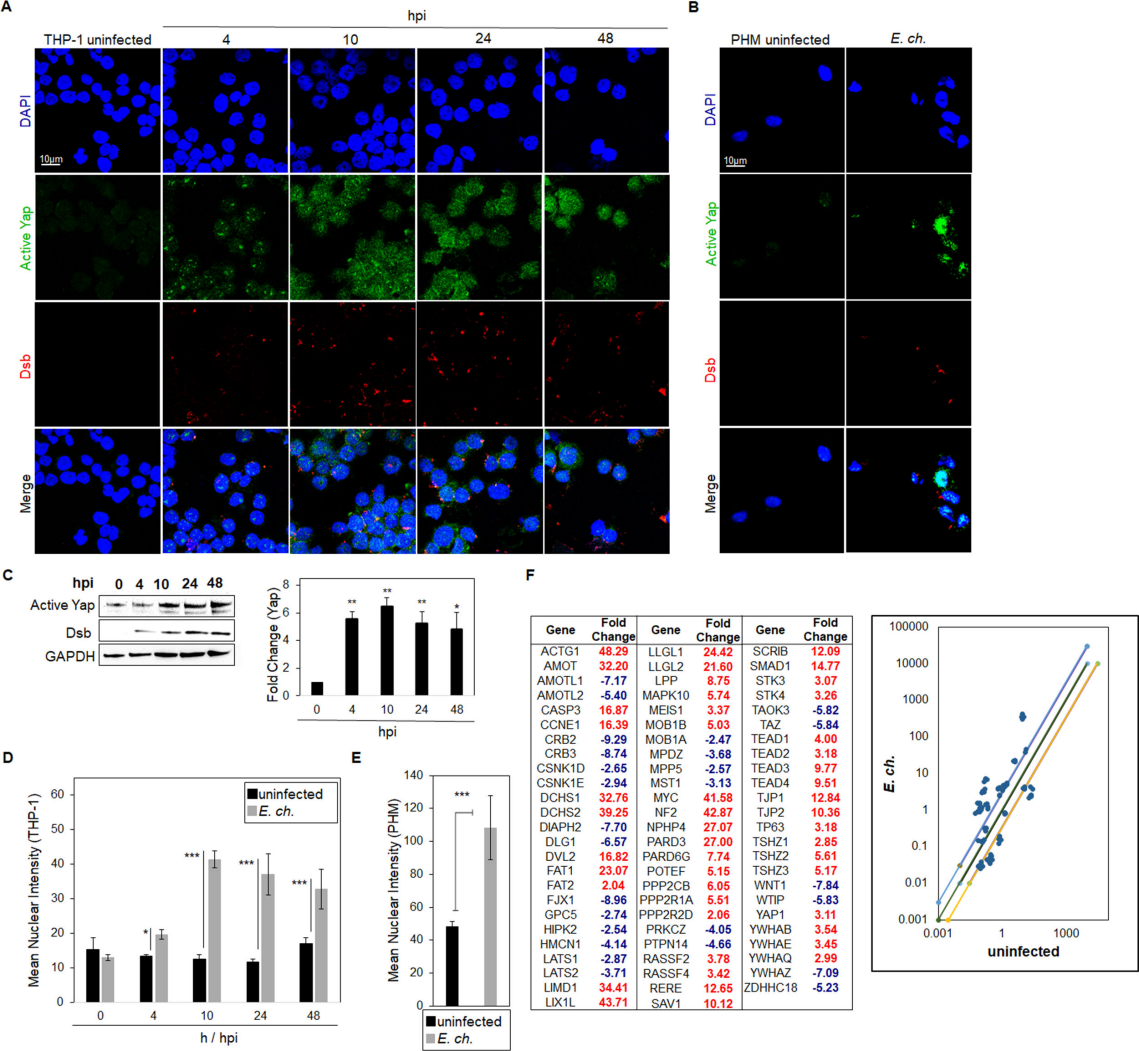
*E. chaffeensis* activates Yap and Hippo gene expression. (**A**) Confocal immunofluorescence micrographs showing temporal Yap activation (green) at 0, 4, 10, 24, and 48 h post-infection (hpi) in *E. chaffeensis*-infected THP-1 cells (MOI 100). Anti-Dsb antibody (red) confirms *E. chaffeensis* infection (scale bar = 10 μM). (**B**) Confocal immunofluorescence micrographs showing Yap activation (green) in uninfected and *E. chaffeensis-*infected (10 h) PHMs (MOI 100). Anti-Dsb antibody (red) confirms *E. chaffeensis* infection (scale bar = 10 μM). (**A and B**) Experiments were performed with three biological and technical replicates. Randomized areas/slides (*n* = 10) were selected to detect active Yap. (**C**) Western blot analysis depicting active Yap levels at 0, 4, 10, 24, and 48 hpi with GAPDH as a loading control. Anti-Dsb antibody demonstrates *E. chaffeensis* infection. The bar graph (right) represents densitometry values of Western blots normalized to GAPDH. Western blots were performed with three biological and technical replicates for *t*-test analysis. Data are represented as means ± SD (*P < 0.05; **P < 0.01). (**D and E**) Intensity graphs demonstrate the mean nuclear accumulation of active Yap in THP-1 cells and PHMs, respectively. Analysis was performed using ImageJ to determine the mean gray value from randomized areas/slides (*n* = 10), and data are shown as mean ± SD (*P < 0.05; ***P < 0.001). (**F**) The table represents the normalized expression of significantly regulated Hippo array genes between *E. chaffeensis-*infected and uninfected cells at 24 h. The scatterplot represents the expression of all Hippo array genes. The top and bottom lines depict a twofold upregulation or downregulation, respectively, compared to an uninfected control. Scatterplots are representative of three (*n* = 3) biological and technical replicates.

Hippo deactivation mediated by Wnt ligand engagement of the Fzd5 receptor results in Yap activation and nuclear translocation, where it binds the transcription factor TEAD to regulate Hippo gene targets (12, 20, 41 - 43). Recent studies demonstrate that *E. chaffeensis* directly interacts with the Fzd5 receptor to activate β-catenin (7). To investigate whether *E. chaffeensis* activates Yap via Hippo-Wnt ligand-receptor crosstalk, confocal microscopy was used to detect active Yap in the nucleus of infected THP-1 cells within 4 h post-infection (hpi). Moreover, significant nuclear accumulation of active Yap was observed over 48 hpi compared to uninfected controls based on intensity graphs (Fig. 1A, C, and D). Further, active Yap accumulated in the nucleus of *E. chaffeensis*-infected primary human monocytes (PHMs; 10 hpi) compared to the uninfected control (Fig. 1B and E). To examine the role of *E. chaffeensis* in Hippo deactivation (Yap activation), we examined Hippo pathway gene transcription using a human Hippo signaling PCR array (Fig. 1F). Significant activation of Hippo pathway component genes was detected during *E. chaffeensis* infection, with the majority (63%) of Hippo genes being upregulated, including major Hippo and Wnt components *YAP*, *TAZ*, *TEAD1, TEAD2*, *TEAD3*, *TEAD4,* and *DVL2* compared to controls (Fig. 1F).

### TRP120 activation of Yap and Hippo gene targets

**Fig 2:**
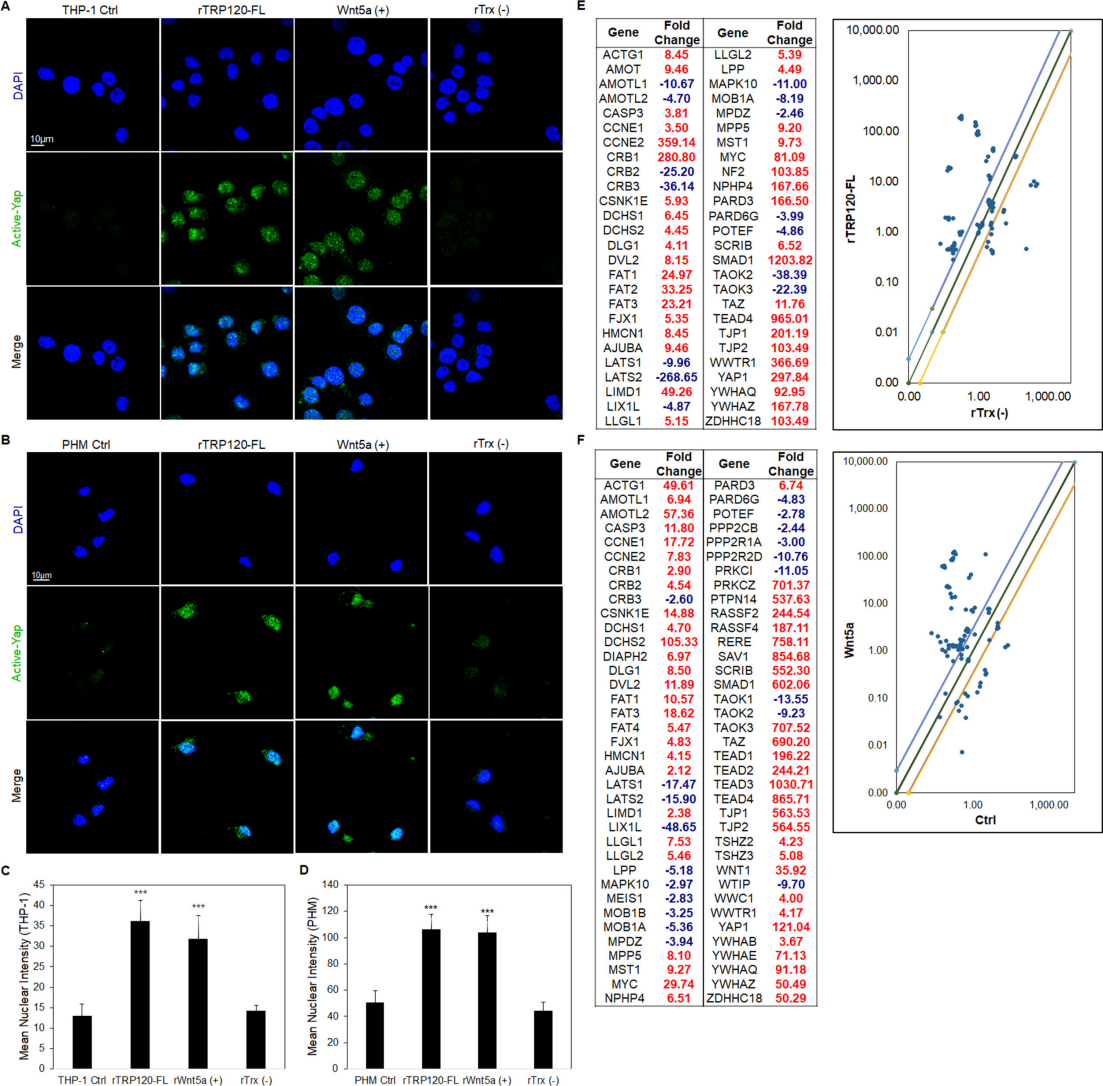
TRP120 activation of Yap and Hippo gene targets. (**A**) Confocal immunofluorescence micrographs demonstrating rTRP120-FL-, rTrx- (−), rWnt5a-treated (+) (1 μg/mL), or untreated (control) THP-1 cells stained with active Yap antibody (green) 6 h post-treatment (scale bar = 10 μM). (**B**) Confocal immunofluorescence microscopy of untreated (control) or rTRP120-FL-, rTrx- (−), rWnt5a-treated (+) (1 µg/mL) PHMs stained with active Yap antibody (green) 10 hpt (scale bar = 10 μM). (**A and B**) Experiments were performed with three biological and technical replicates. Randomized areas/slides (*n* = 10) were selected to detect active Yap. (**C and D**) Intensity graphs demonstrate the mean nuclear accumulation of active Yap in the respective THP-1 cells and PHMs. Analysis was performed using ImageJ to determine the mean gray value from randomized areas/slides (*n* = 10), and data are shown as mean ± SD (***P < 0.001). (**E**) The table represents significantly regulated Hippo signaling PCR array gene expression in THP-1 cells stimulated with rTRP120-FL (1 µg/mL) after normalization to control cells treated with rTrx (1 μg/mL) at 24 h. The respective normalized expression of rTRP120-FL-regulated Hippo array genes was performed with three biological and technical replicates. (**F**) The table represents significantly regulated Hippo signaling PCR array gene expression in THP-1 cells stimulated with rWnt5a (1 μg/mL) after normalization to dimethylsulfoxide (DMSO)-treated cells (control). The respective normalized expression of rWnt5a-regulated Hippo array genes is representative of three biological replicates. (**E and F**) The scatterplot represents the expression of all Hippo array genes. The top and bottom scatterplot lines depict a twofold upregulation or downregulation, respectively, compared to control. Data are representative of three independent experiments (*n* = 3).

To examine the role of TRP120 in Hippo deactivation, THP-1 cells and PHMs were incubated with recombinant TRP120 protein (rTRP120-FL), and Yap activation was examined using confocal microscopy (Fig. 2A and B). Active Yap accumulated in the nucleus of THP-1 cells (Fig. 2A and C) and PHMs (Fig. 2B and D) at 6 and 10 h post-treatment (hpt), respectively, consistent with Yap activation by recombinant Wnt5a (rWnt5a). To further confirm the role of TRP120 in Hippo regulation, cells were stimulated with rTRP120-FL or rWnt5a for 24 h, and a transcriptional analysis was performed (Fig. 2E and F). Hippo genes (45%) were significantly upregulated compared to the rTrx negative control, including genes important for Hippo and Wnt signaling (*YAP*, *TAZ*, *TEAD4,* and *DVL2*), and 16% were downregulated (Fig. 2E). In comparison, cells treated with rWnt5a had significant transcriptional upregulation of Hippo genes (65%), including *YAP*, *TAZ*, *TEAD1*, *TEAD2, TEAD3, TEAD4, WNT1,* and *DVL2*, and 22% of genes were significantly downregulated (Fig. 2F). Though there were differential expression patterns of genes in TRP120 and Wnt5a-treated cells, we found that 34 Hippo target genes, including *YAP, TAZ, TEAD4,* and *DVL2,* were upregulated in both rTRP120-FL and rWnt5a treatments. Together, these data demonstrated that treatment of THP-1 cells with rTRP120-FL resulted in intracellular Yap accumulation and changes in Hippo pathway gene transcript levels that were similar, but not identical, to those observed after incubation with rWnt5.

### TRP120-Wnt-SLiM inactivates Hippo signaling and activates Yap

**Fig 3:**
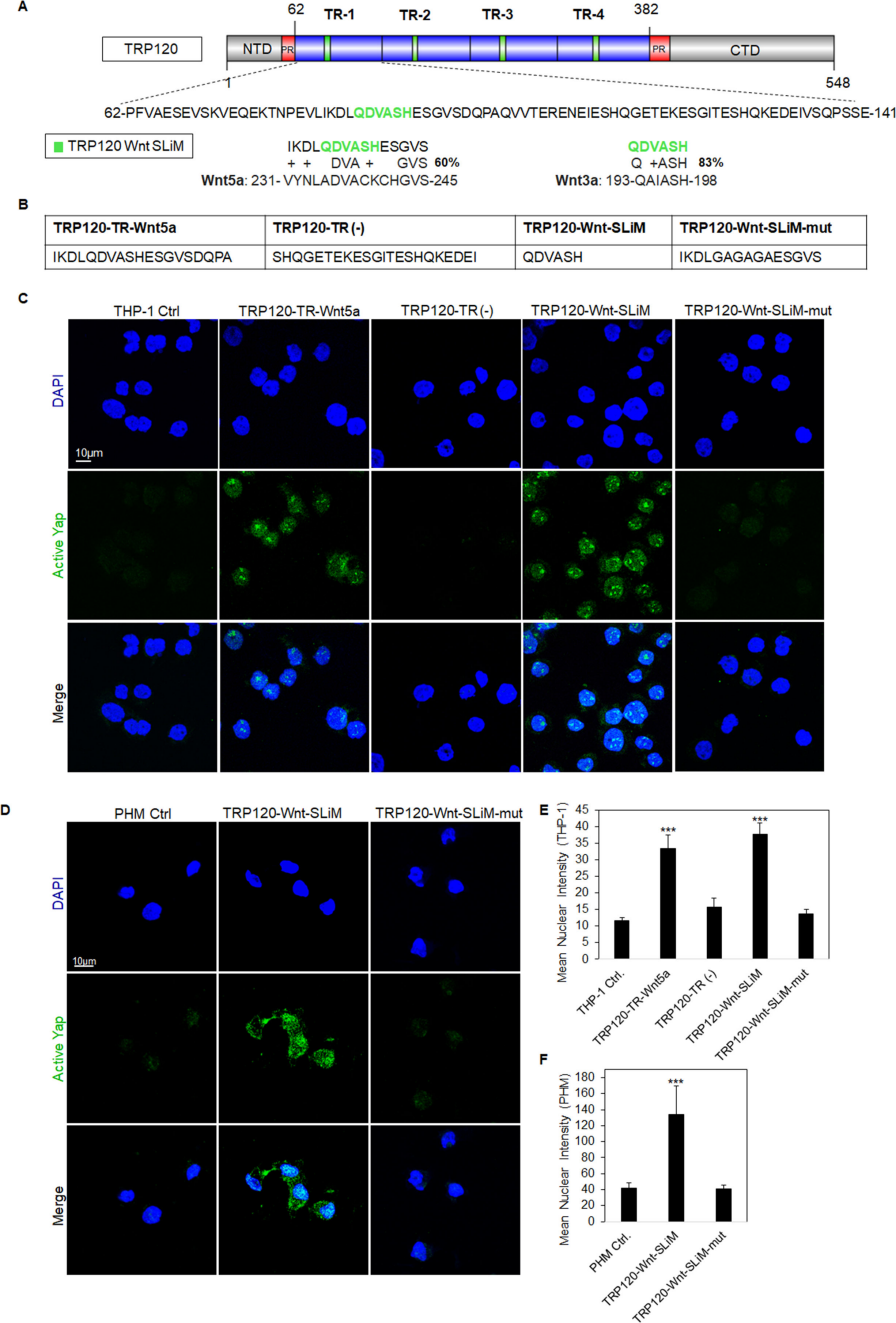
TRP120-Wnt-SLiM inactivates Hippo signaling and activates Yap. (**A**) Schematic representation of TRP120 showing domain organization: N-terminal (NTD), C-terminal (CTD), tandem repeat (TR1–4; 80 aa each), and partial repeat (PR) domain (44). A six amino acid short linear motif (SLiM) of high sequence similarity was identified using NCBI BLAST between the TRP120 TR and Wnt3a/Wnt5a ligands (activators of Yap) amino acid sequences. The complete amino acid sequence of one TR is shown, with homologous Wnt SLiM identified in green and percent homology to the right of the sequence. (**B**) The table displays the various TRP120 peptide amino acid sequences used in the TRP120-Wnt-SLiM study. TRP120-Wnt-SLiM represents the homology sequence identified through BLAST. TRP120-Wnt-SLiM-mut contains glycine and alanine substitutions in the Wnt-SLiM region and is used as a negative control. TRP120-TR-Wnt5a is a 19-amino acid sequence that contains the identified TRP120-Wnt homology sequence. TRP120-TR (−) is a sequence within TRP120-TR that does not contain the defined TRP120-Wnt homology sequence. (**C**) Confocal immunofluorescence microscopy of untreated (−) or peptide-treated THP-1 cells (1 μg/mL). THP-1 cells were stained with active Yap antibody, and the micrograph shows increased levels of active Yap (green) in TRP120-TR-Wnt5a and TRP120-Wnt-SLiM-treated, but not in untreated, TRP120-TR (−) or TRP120-Wnt-SLiM-mut-treated THP-1 cells (6 hpt) (scale bar = 10 μM). (**D**) Confocal immunofluorescence microscopy of untreated or SLiM/SLiM mutant peptide-treated PHMs (10 h). The TRP120-Wnt-SLiM sequence upregulates active Yap (green) in PHMs, but the corresponding mutant sequence does not (scale bar = 10 μM). (**C and D**) Experiments were performed with three biological and technical replicates. Randomized areas/slides (*n* = 10) were selected to detect active Yap nuclear translocation. (**E and F**) Intensity graphs demonstrate the mean nuclear accumulation of active Yap in the respective THP-1 cells and PHMs. Analysis was performed using ImageJ to determine the mean gray value from randomized areas/slides (*n* = 10). Data are represented as means ± SD (***P < 0.001).

TRP120 contains a tandem repeat domain (TRD), with four tandem repeats, flanked by N- and C-terminal domains. Various TRP120 SLiMs have been reported within the TRD and C-terminus that are relevant to *E. chaffeensis* pathobiology, including posttranslational modification motifs, DNA-binding motifs, and ubiquitin ligase catalytic motifs (7). We previously reported that *E. chaffeensis* TRP120 TRD utilizes SLiMs to regulate Wnt, Notch, and Hedgehog signaling pathways (7, 9, 10). The discovery of a TRP120-Wnt-SLiM that activates Wnt signaling was previously reported, and homology was identified between TRP120 and Wnt5a (7). However, based on a revised BLAST analysis of TRP120 with both Wnt5a and Wnt3a (Fig. 3A), we identified a shorter Wnt SLiM (QDVASH) shared by both ligands (60% and 83% similarity, respectively) within the previously identified TRP120-Wnt-SLiM (IKDLQDVASHESGVSDQ).

To investigate Wnt SLiM activation of Yap, THP-1 cells were treated for 6 h with two peptides that contained the following sequences: TRP120-Wnt-SLiM (6 aa) and TRP120-TR-Wnt5a (19 aa); and two control peptides that did not contain Wnt SLiM: TRP120-Wnt-SLiM-mut (15 aa; glycine/alanine substitutions) and TRP120-TR (−) (22 aa; TR sequence null of Wnt SLiM) (Fig. 3B) and Yap activation was determined (Fig. 3C and E). Both TRP120-TR-Wnt5a and TRP120-Wnt-SLiM treatments stimulated greater fluorescent intensity reflective of Yap levels compared to TRP120-TR (−) and TRP120-Wnt-SLiM-mut controls (Fig. 3E). Similarly, TRP120-Wnt-SLiM treatment significantly stimulated active Yap in PHMs (10 hpt) compared to TRP120-Wnt-SLiM-mut (Fig. 3D and F). Additionally, to determine whether the newly defined TRP120-Wnt SLiM activates Wnt signaling, we treated THP-1 cells with TRP120-Wnt-SLiM and TRP120-Wnt-SLiM-mut and measured active β-catenin nuclear accumulation (Fig. S1A). TRP120-Wnt-SLiM was able to significantly stimulate active β-catenin consistent with *E. chaffeensis* and Wnt5a, confirming that the 6 aa Wnt SLiM was completely responsible for Wnt signaling activation and the amino acids flanking the 6 aa Wnt SLiM were not significant.

Further, we treated THP-1 cells with a single amino acid TRP120-Wnt-SLiM histidine deletion mutant (QDVAS) and observed no significant activation of Yap or β-catenin, indicating that the 6 amino acid TRP120-Wnt-SLiM containing histidine is essential for activation (Fig. S2).

### TRP120-Wnt-SLiM concentration-dependent Hippo gene activation

**Fig 4:**
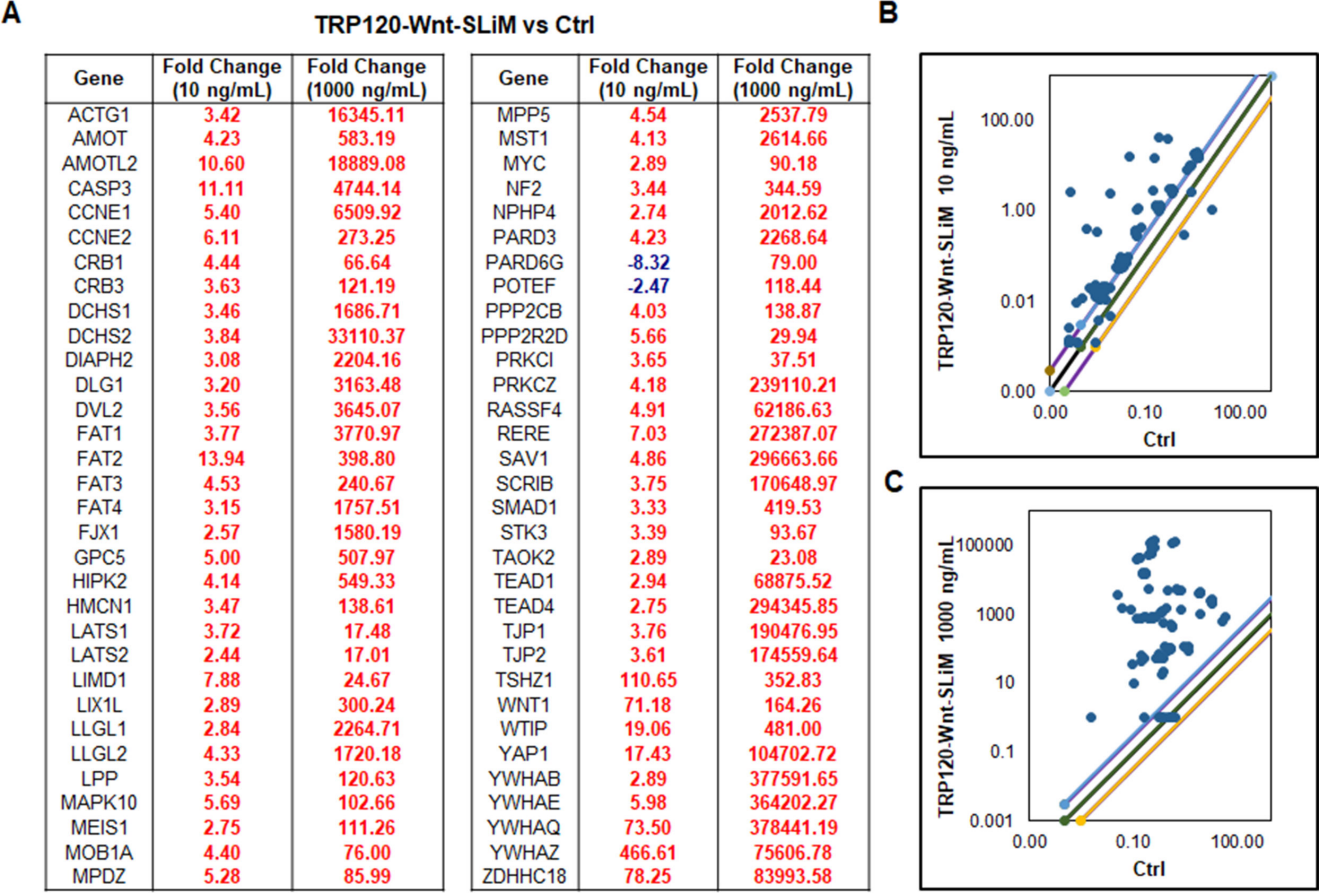
TRP120-Wnt-SLiM concentration-dependent Hippo gene activation. (**A**) Hippo signaling PCR array was used for the analysis of the expression of 84 Hippo genes. THP-1 cells were treated with TRP120-Wnt-SLiM (10 and 1,000 ng/mL) or left untreated (negative control) and harvested at 24 h. The tables represent significant fold changes in gene expression in TRP120-Wnt-SLiM-treated cells compared to untreated cells at respective concentrations. Data represent three (*n* = 3) biological replicates. (**B and C**) The scatterplots represent the expression of all Hippo array genes. The top and bottom scatterplot lines depict a twofold upregulation or downregulation, respectively, compared to control. Scatterplots are representative of three independent experiments (*n* = 3).

To investigate if TRP120-Wnt-SLiM regulates Hippo target genes, THP-1 cells were treated with TRP120-Wnt-SLiM (10 or 1,000 ng/mL), and significant Hippo gene activation was detected in a concentration-dependent manner (Fig. 4A through C). TRP120-Wnt-SLiM influenced Hippo gene expression, including Hippo and Wnt target genes *YAP*, *WNT1*, *DVL2*, *TEAD1*, and *TEAD4,* consistent with *E. chaffeensis*, TRP120-, and WNT5a (10 ng/mL). Moreover, all Hippo genes were upregulated in response to a 100-fold higher TRP120-Wnt-SLiM concentration (1,000 ng/mL).

### Hippo co-activator and transcription factors influence infection

**Fig 5:**
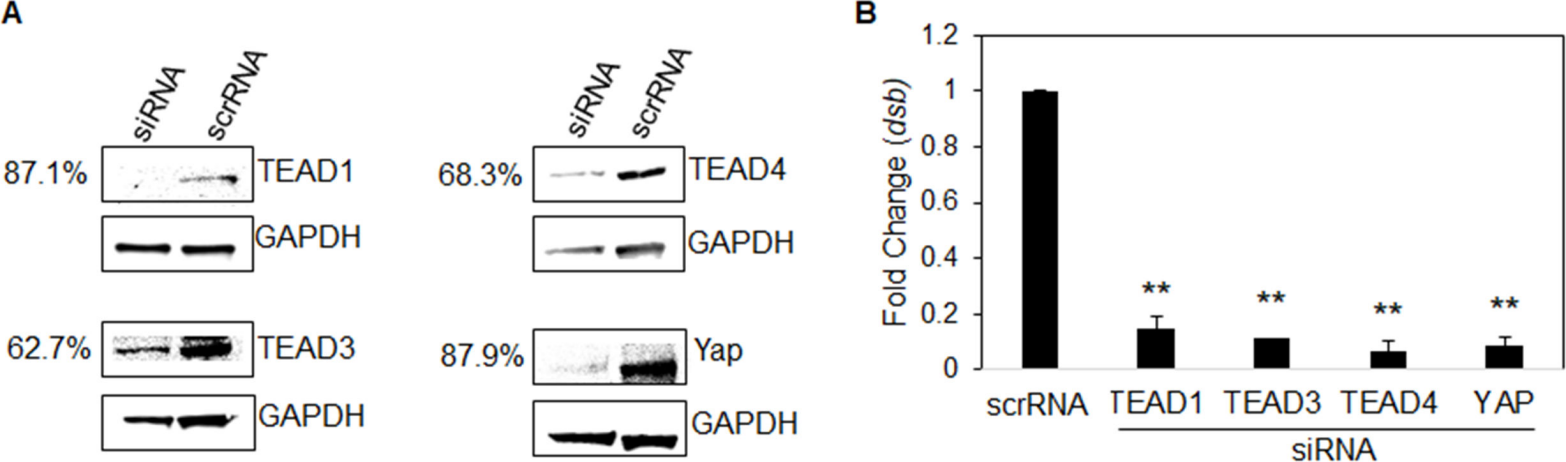
Hippo co-activator and transcription factors influence infection. (**A**) Western blots depict the knockdown efficiency of small interfering RNA-transfected (siRNA) THP-1 cells, with scrambled siRNA (scrRNA) transfected THP-1 cells as a control from whole-cell lysates (24 hpt). siRNA knockdown (%) indicates the total percent knockdown of protein of interest relative to the control, normalized to GAPDH. (**B**) THP-cells (24 hpt) were infected with *E. chaffeensis* (MOI 100) and harvested at 24 hpi. Infected scrRNA cells are represented as a positive control. qPCR amplification of the ehrlichial disulfide bond formation protein (*dsb*) gene was used to quantify *E. chaffeensis* infection. siRNA knockdown of Hippo transcription components TEAD1-4 and Yap significantly inhibits *E. chaffeensis* infection in THP-1-transfected cells. All knockdowns were performed with three biological and technical replicates for *t*-test analysis. Data are represented as mean ± SD (**P < 0.01).

Although the Hippo pathway is widely recognized for its role in embryogenesis and tumorigenesis, it also plays a key role in regulating apoptosis, which is crucial for successful ehrlichial intracellular infection (4, 10, 16, 17, 22). To determine whether *E. chaffeensis* survival depends on Hippo transcriptional components, we used RNAi to individually silence genes for *YAP*, *TEAD1*, *TEAD3,* and *TEAD4* (Fig. 5A). Ehrlichial load was significantly reduced in all transfection groups 24 h post-RNAi transfection compared to the scramble siRNA-transfected controls (Fig. 5B).

### A TRP120-Wnt-SLiM antibody blocks Yap activation

**Fig 6:**
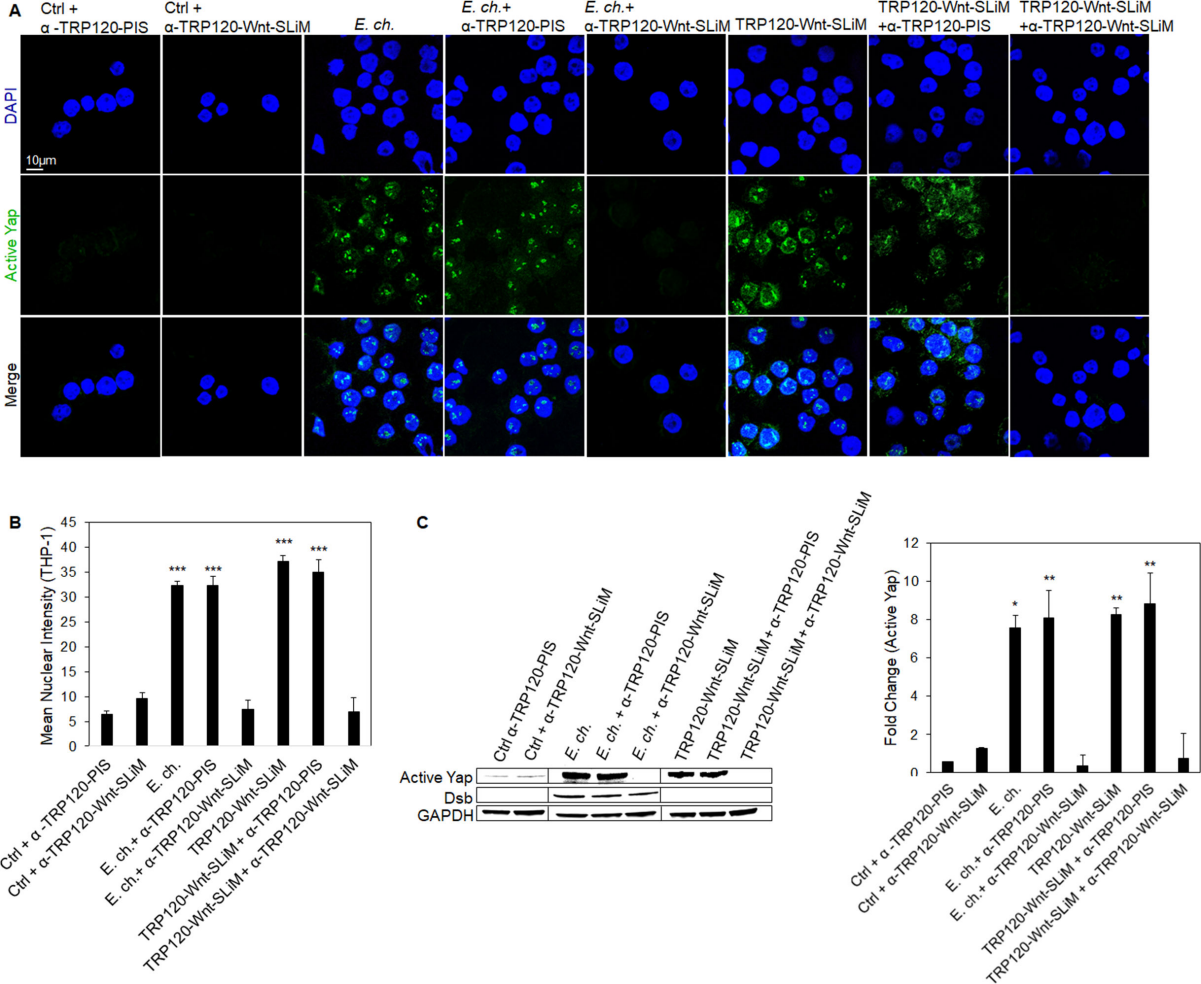
A TRP120-Wnt-SLiM antibody blocks Yap activation. (**A**) *E. chaffeensis* (MOI 100) and TRP120-Wnt-SLiM (1 μg/mL) were incubated with α-TRP120-Wnt-SLiM (targets TRP120 sequence DLQDVASHESGVSDQPAQV) or α-TRP120-PIS (neg ctrl) (1.5 μg/mL) for 1 h or overnight, respectively, before incubation with THP-1 cells. THP-1 cells were harvested at 6 hpt, immunostained with active Yap antibody (green), and visualized by confocal fluorescence microscopy (scale bar = 10 μM). Randomized areas/slides (*n* = 10) were selected to detect active Yap nuclear translocation. (**B**) The intensity graph demonstrates the mean nuclear accumulation of active Yap in respective THP-1 cells. Analysis was performed using ImageJ to find the mean gray value from randomized areas/slides (*n* = 10). (**C**) Western blot analysis of treatment groups with GAPDH as a loading control with a bar graph of Western blot analyzed from densitometry values normalized to GAPDH (**A–C**); α-TRP120-Wnt-SLiM inhibits active Yap upregulation in cells with *E. chaffeensis* or TRP120-Wnt-SLiM compared to α-TRP120-PIS. Untreated cells were incubated with α-TRP120-Wnt-SLiM or α-TRP120-PIS as negative controls. Experiments were performed with three biological and technical replicates, and significance was determined through *t*-test analysis. Data are represented as means ± SD (*P < 0.05; **P < 0.01; ***P < 0.001).

To further elucidate the role of TRP120-Wnt-SLiM during *E. chaffeensis* infection, we investigated whether blocking *E. chaffeensis* infection or the TRP120-Wnt-SLiM with a TRP120-Wnt-SLiM-targeted antibody would inhibit Yap activation. *E. chaffeensis-*infected and TRP120-Wnt-SLiM-treated cells in the presence of α-TRP120-Wnt-SLiM demonstrated a significant reduction in active Yap relative to *E. chaffeensis-*infected and TRP120-Wnt-SLiM-treated cells in the presence of α-TRP120-PIS antibody (control) (Fig. 6A through C).

### Hippo deactivation is Fzd5 receptor dependent

**Fig 7:**
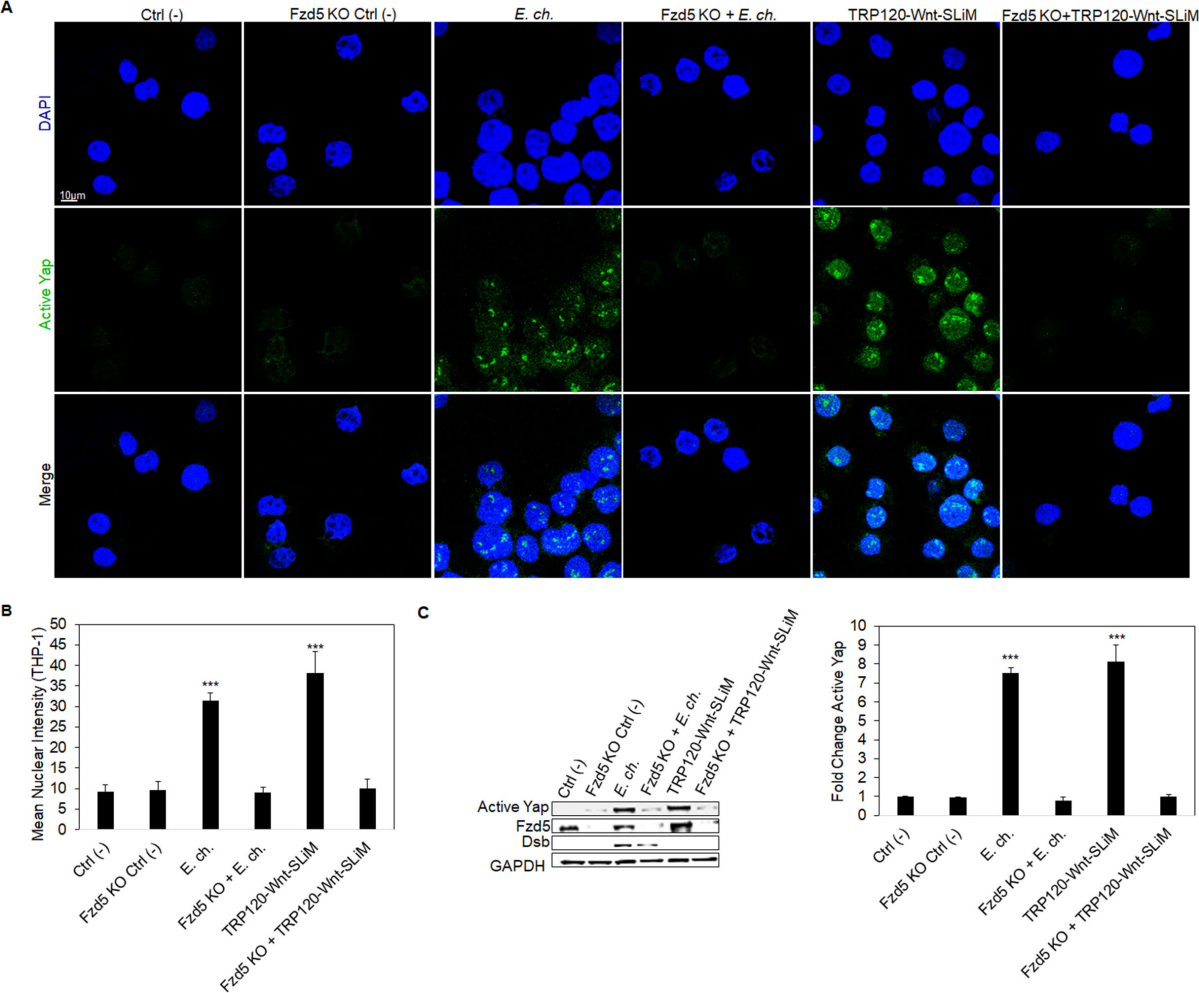
Hippo deactivation is Fzd5 receptor-dependent. (**A**) Confocal immunofluorescence microscopy of untreated (−), *E. chaffeensis*-infected (MOI 100), or TRP120-Wnt-SLiM-treated (1 μg/mL) THP-1 cells compared to Fzd5 receptor KO THP-1 cells. THP-1 cells and Fzd5 receptor KO THP-1 cells were harvested 6 hpt, immunostained with active Yap antibody (green), and visualized by confocal fluorescence microscopy (scale bar = 10 μM). Randomized areas/slides (*n* = 10) were selected to detect active Yap nuclear translocation. (**B**) The intensity graph demonstrates the mean nuclear accumulation of active Yap in respective THP-1 cells. Analysis was performed using ImageJ to determine the mean gray value from randomized areas/slides (*n* = 10). (**C**) Western blot analysis of treatment groups to determine active Yap, Fzd5, and Dsb levels with GAPDH as a loading control. Western blot bar graph was analyzed from densitometry values normalized to GAPDH. (**A–C**) Experiments were performed with three biological and technical replicates, and significance was determined through *t*-test analysis. Data are represented as means ± SD (***P < 0.001).

During Hippo-Wnt receptor crosstalk, Wnt5a and Wnt3a ligands bind the Fzd5 receptor to deactivate Hippo signaling and activate Yap to engage Hippo gene transcription. We previously demonstrated that *E. chaffeensis* TRP120 TRD directly binds the Fzd5 receptor (7). To determine the basis of this interaction regarding Hippo signaling, Fzd5 receptor knockout (KO) cells were used to determine the role of the Fzd5 receptor in Yap activation. Fzd5 receptor KO or normal THP-1 cells (control) were infected with *E. chaffeensis* or treated with TRP120-Wnt-SLiM. Fzd5 receptor KO cells exhibited no significant Yap activation compared to the control (Fig. 7A through C). Similarly, we determined that there was significant deactivation of β-catenin in THP-1 Fzd5 receptor KO cells infected with *E. chaffeensis* or treated with TRP120-Wnt-SLiM, revealing that *E. chaffeensis* interacts with Fzd5 receptor to activate Wnt signaling (Fig. S1B). However, unlike Yap, there was significant activation of β-catenin compared to control cells, likely due to the contribution of other Fzd receptors known to interact with TRP120 (7).

### Hippo target gene *SLC2A1* is upregulated during *E. chaffeensis* infection

**Fig 8:**
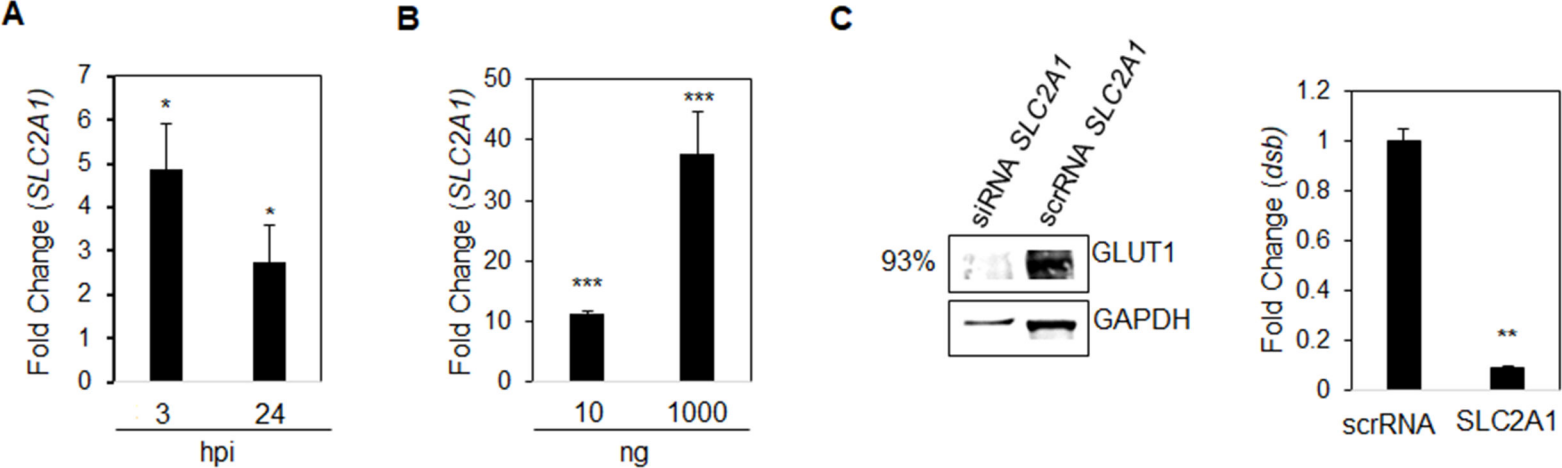
Hippo target gene *SLC2A1* is upregulated during *E. chaffeensis* infection. (**A and B**) Real-time qPCR analysis of anti-apoptotic regulator, *SLC2A1*, normalized to *GAPDH* during *E. chaffeensis* infection (MOI 100) at 3 and 24 hpi (**A**) and TRP120-Wnt-SLiM treatment (10 ng and 1,000 ng) (**B**), demonstrating *SLC2A1* transcriptional activation. Uninfected and untreated controls were used to calculate fold changes. (**C**) Western blots depict the knockdown efficiency of small interfering RNA-transfected (siRNA) THP-1 cells, with scrambled siRNA (scrRNA) transfected THP-1 cells as a control from whole-cell lysates harvested at 24 h post-transfection (as described in Fig. 5). siRNA knockdown (%) indicates the total percent knockdown of *SLC2A1* relative to control, normalized to GAPDH. qPCR amplification of the ehrlichial disulfide bond formation protein (*dsb*) gene was used to quantify *E. chaffeensis* infection (MOI 100) at 24 hpi. Infected scrRNA cells are represented as a positive control. (**A and C**) Experiments were performed with three biological and technical replicates, and significance was determined through *t*-test analysis. Data are represented as means ± SD (*P < 0.05; **P < 0.01; ***P < 0.001).

To understand the basis and downstream effects of Hippo regulation during *E. chaffeensis* infection, the Hippo target gene and the anti-apoptotic *SLC2A1* were investigated. *SLC2A1* encodes the glucose transporter GLUT1, which is necessary for preventing apoptosis through the Yap-GLUT1-BCL-xL axis (19, 23, 26, 33, 45). During *E. chaffeensis* infection, significant upregulation of *SLC2A1* was detected at 3 and 24 hpi (Fig. 8A). Further, TRP120-Wnt-SLiM upregulated *SLC2A1* in a concentration-dependent manner at 6 hpt (Fig. 8B). To determine whether *E. chaffeensis* infection relies on *SLC2A1* for survival, we used RNAi to silence *SLC2A1* in THP-1 cells. The ehrlichial load was significantly reduced (24 hpi) in *SLC2A1*-siRNA-transfected cells compared to the scramble control transfected cells (Fig. 8C).

### *E. chaffeensis* TRP120-Wnt-SLiM-mediated regulation of GLUT1, BCL-xL, and Bax

**Fig 9:**
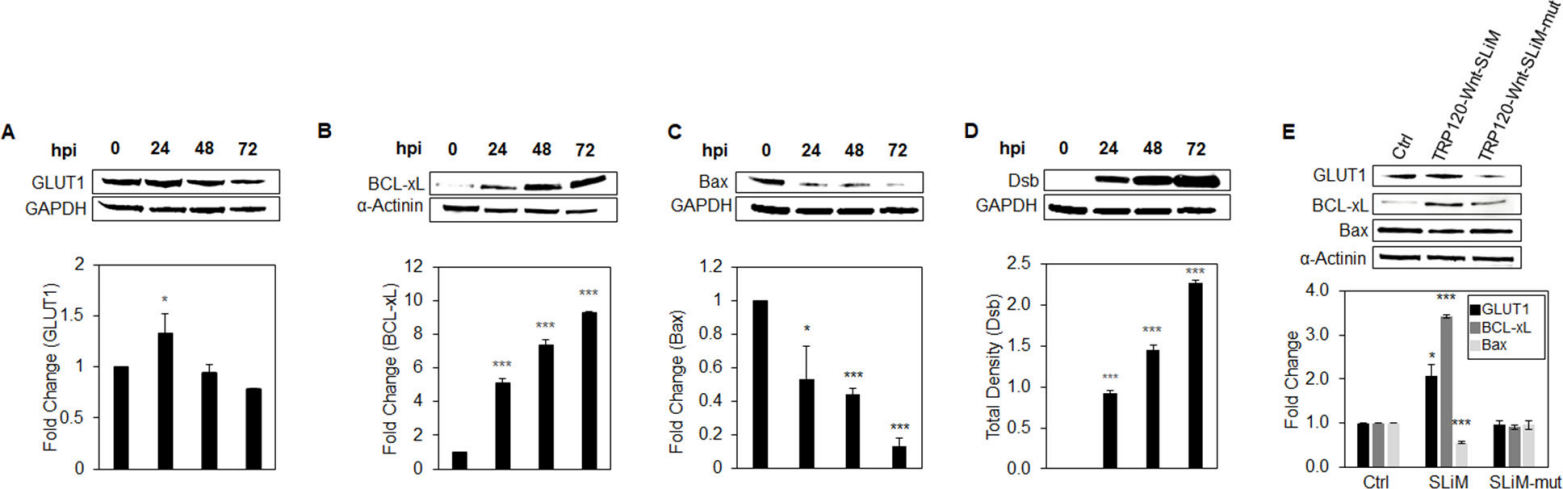
*E. chaffeensis* TRP120-Wnt-SLiM-mediated regulation of GLUT1, BCL-xL, and Bax. (**A–C**) Western blot analysis of GLUT1, BCL-xL, and Bax levels during *E. chaffeensis* infection at 0, 24, 48, and 72 hpi. GAPDH or α-actinin was used as a loading control and Dsb as an infection control (**D**). (**E**) GLUT1, BCL-xL, and Bax levels during TRP120-Wnt-SLiM, TRP120-Wnt-SLiM-mut-treated (1 μg/mL) and untreated THP-1 cells (24 hpt) with GAPDH as a loading control. (**C and D**) The blots were obtained from the same Western blot membrane. As a result, the GAPDH in these panels is identical. (**A–E**) Bar graphs depict Western blot densitometry values normalized to GAPDH or α-actinin. Experiments were performed with three biological and technical replicates, and significance was determined through *t*-test analysis. Data are represented as mean ± SD (*P < 0.05; ***P < 0.001).

It is well documented that Hippo signaling promotes cell proliferation and prevents cell apoptosis through the Yap-GLUT1-BCL-xL axis (19, 23, 26, 33, 45). Further, BCL-xL is involved in the inhibition of mitochondria-mediated pro-death pathway by directly inhibiting Bax and subsequent caspase activation (45 - 47). Based on our results, we hypothesized that *E. chaffeensis* deactivates Hippo signaling and activates Yap to increase GLUT1 and BCL-xL and decrease Bax levels. To examine this question, THP-1 cells were infected with *E. chaffeensis* or treated with TRP120-Wnt-SLiM and TRP120-Wnt-SLiM-mut. *E. chaffeensis* and TRP120-Wnt-SLiM significantly increased GLUT1 and BCL-xL and decreased Bax levels compared to controls, consistent with a Yap-dependent anti-apoptotic profile induced by *E. chaffeensis* (Fig. 9A through D).

### TRP120-Wnt-SLiM-mediated regulation of GLUT1, BCL-xL, and Bax during Yap inhibition

**Fig 10:**
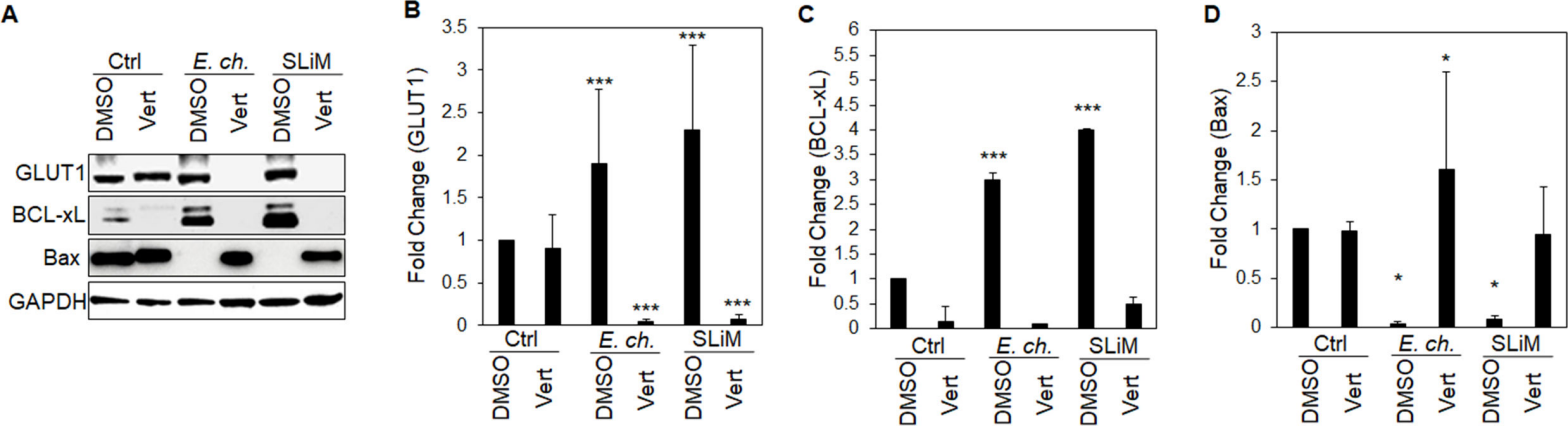
TRP120-Wnt-SLiM-mediated regulation of GLUT1, BCL-xL, and Bax during Yap inhibition. (**A–D**) Western blot analysis of GLUT1, BCL-xL, and Bax levels during *E. chaffeensis* infection or TRP120-Wnt-SLiM treatment (1 μg/mL). THP-1 cells in the presence of the Yap inhibitor Verteporfin (Vert) were collected at 24 h with GAPDH as a loading control. (**B**) GLUT1 protein expression significantly reduces in the presence of Verteporfin and significantly increases in the presence of DMSO (control) during *E. chaffeensis* infection and SLiM treatment. (**C**) BCL-xL levels significantly increase during *E. chaffeensis* infection and in TRP120-Wnt-SLiM-treated cells in the presence of DMSO (control) compared to Verteporfin. (**D**) Bax levels significantly increase during infection in the presence of Verteporfin compared to DMSO (control). (**B–D**) Bar graphs depict Western blot densitometry values normalized to GAPDH. Experiments were performed with three biological and technical replicates, and significance was determined through *t*-test analysis. Data are represented as mean ± SD (*P < 0.05; ***P < 0.001).

Our results support the importance of the anti-apoptotic Yap-GLUT1-BCL-xL axis during infection. Further, we hypothesized that *E. chaffeensis* deactivates Hippo to regulate GLUT1, BCL-xL, and Bax. To test this hypothesis, we used a Yap inhibitor (Verteporfin) to determine whether *E. chaffeensis* infection or TRP120-Wnt-SLiM regulated GLUT1, BCL-xL, and Bax levels during Yap inhibition. During infection, there was a significant reduction in GLUT1 in the presence of Verteporfin, demonstrating that *E. chaffeensis* depends on Yap activation to increase GLUT1 (Fig. 10B). Additionally, during Verteporfin treatment, BCL-xL levels were insignificant during *E. chaffeensis* infection compared to the control (Fig. 10C). Further, Bax levels significantly increased during *E. chaffeensis* infection in the presence of Verteporfin compared to the control (Fig. 10D).

### Yap inhibition induces an apoptotic profile during *E. chaffeensis* infection

**Fig 11:**
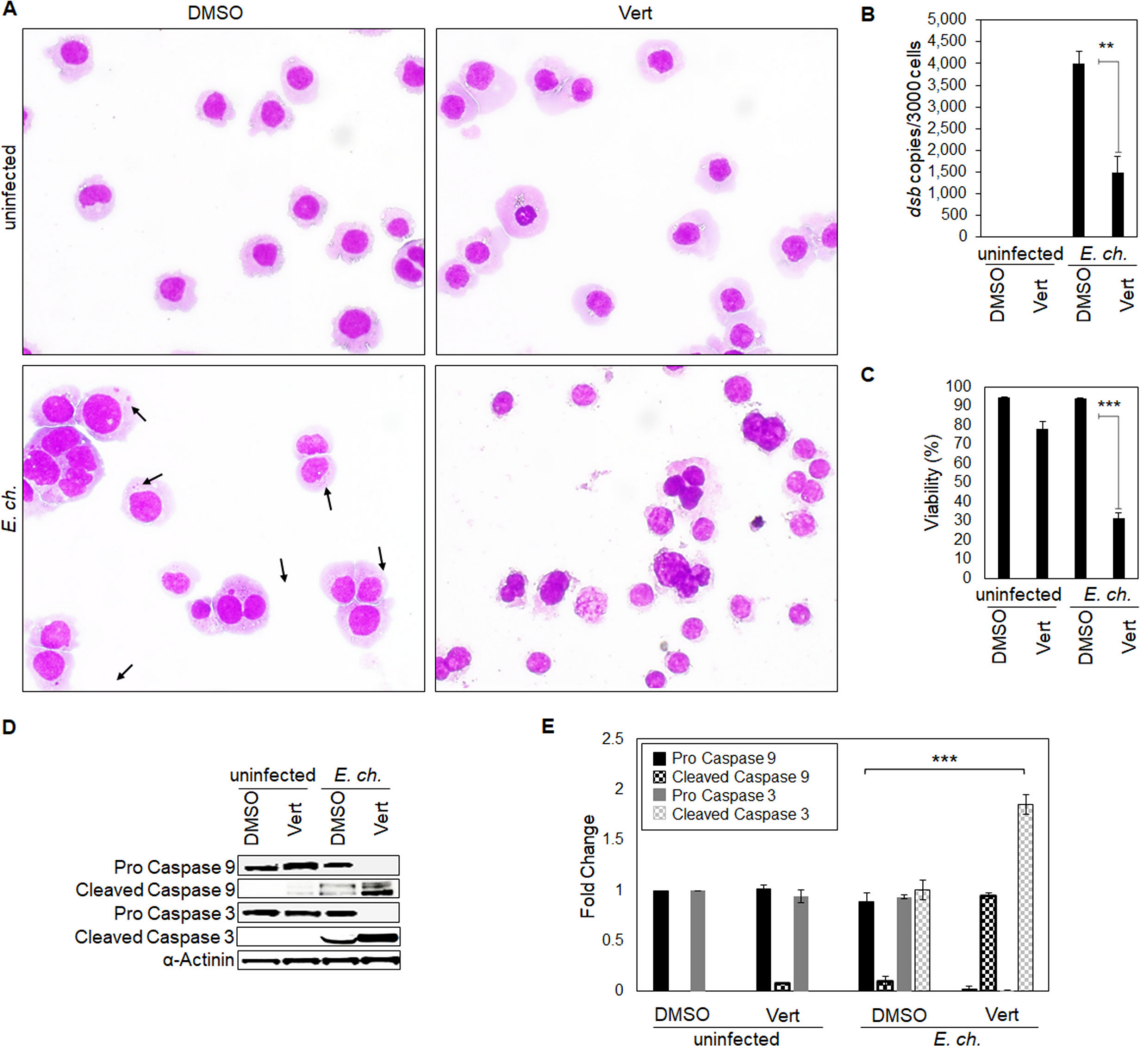
Yap inhibition induces an apoptotic profile during *E. chaffeensis* infection. (**A**) Brightfield micrographs showing effects of DMSO or Hippo inhibitor Verteporfin on uninfected and *E. chaffeensis*-infected THP-1 (MOI 50) cells prepared using Diff-Quick staining. *E. chaffeensis*-infected or uninfected THP-1 cells were treated with DMSO or Verteporfin (7 μg/mL) and collected 24 h later. *E. chaffeensis-*infected THP-1 cells treated with Verteporfin undergo cytoplasmic condensation (a precursor to apoptosis), but other treatment groups do not (arrows point to morulae). (**B**) Bar graph showing fold change in *E. chaffeensis* infection for each treatment group. Ehrlichial loads were determined using qPCR measurement of *dsb* copy and normalized to *GAPDH. E. chaffeensis* infection significantly declines in the presence of Verteporfin. (**C**) Bar graphs showing cell viability for each treatment group. Cell viability was determined using the Cellometer Mini bright field automated cell counter and the pattern-recognition assay. Cell viability significantly declines in the presence of Verteporfin during *E. chaffeensis* infection. (**D and E**) Western blot analysis of Caspase-9 and -3 levels for each group with α-actinin as a loading control. Pro-Caspase-9 and -3 levels significantly decreased while cleaved Caspase-9 and -3 levels significantly increased during *E. chaffeensis* infection in the presence of Verteporfin compared to DMSO control. Bar graphs depict Western blot densitometry values normalized to α-actinin. Experiments were performed with three biological and technical replicates, and significance was determined through *t*-test analysis. Data are represented as means ± SD (*P <0.05; **P <0.01; ***P <0.001).

Our results support the importance of the anti-apoptotic Yap-GLUT1-BCL-xL axis during infection and demonstrate that *E. chaffeensis* infection and TRP120-Wnt-SLiM engage the Hippo pathway to regulate GLUT1, BCL-xL, and Bax. Further, we hypothesized that *E. chaffeensis* regulates the anti-apoptotic Yap-GLUT1-BCL-xL axis during infection to prevent subsequent Caspase-9 and -3 activation and intrinsic apoptosis. To test this hypothesis, *E. chaffeensis*-infected and uninfected THP-1 cells were treated with the Yap inhibitor Verteporfin or DMSO (Fig. 11A). *E. chaffeensis-*infected Verteporfin-treated cells demonstrated a significant increase in cytoplasmic condensation (a precursor to apoptosis) at 24 hpi compared to uninfected Verteporfin-treated cells and *E. chaffeensis*-infected and uninfected DMSO-treated cells, supporting the conclusion that *E. chaffeensis* activates Yap to prevent apoptosis. Additionally, ehrlichial survival was significantly reduced in the presence of Verteporfin compared to the control (DMSO) (Fig. 11B). Further, cell viability significantly decreased in *E. chaffeensis-*infected cells treated with Verteporfin (Fig. 11C). To define a direct mechanism by which *E. chaffeensis* activates Yap to prevent apoptosis, we evaluated levels of pro- and cleaved Caspase-9 and -3 during infection in the presence of Verteporfin (Fig. 11D through E). *E. chaffeensis-*infected cells treated with inhibitor showed a significant decrease in pro-Caspase-9 and -3 levels, while cleaved Caspase-9 and -3 levels significantly increased during *E. chaffeensis* infection in the presence of Verteporfin compared to DMSO-treated *E. chaffeensis*-infected cells (control).

## DISCUSSION

Based on the premise of Hippo-Wnt crosstalk and the regulation of Wnt signaling by the TRP120-Wnt-SLiM, we sought to identify whether the TRP120-Wnt-SLiM deactivates Hippo, leading to Yap activation. Indeed, we reveal that TRP120-Wnt-SLiM regulates Hippo signaling and identifies the downstream effects directed at inhibiting intrinsic host-cell apoptosis. *E. chaffeensis* contains a Wnt-SLiM and depends on the Wnt-Fzd5 receptor to activate the transcription co-activator Yap, which promotes a significant upregulation in genes critical for Hippo and Wnt signaling. This is the first report of a single eukaryotic SLiM mimic in bacteria that can regulate multiple conserved signaling pathways. This study also reveals a novel strategy utilized by obligate intracellular bacteria to extend host cell lifespan and highlights the importance of pathogen utilization of eukaryotic cellular signaling motifs for reprogramming the host cell to promote infection.

Although Hippo signaling has been studied during viral infection, little is known regarding Hippo signaling during bacterial infection. We investigated whether *E. chaffeensis* regulates Hippo signaling during infection. Indeed, we confirmed that infection induces Yap activation and transcriptional induction of Hippo pathway genes. Although there was differential expression of Hippo pathway genes in TRP120 and Wnt5a-treated cells, we discovered that many Hippo target genes were upregulated by both TRP120 and Wnt5a, which supports TRP120 mimicry of Wnt5a. Some differences between TRP120 and Wnt5a were expected since the biological functions of various Wnt ligands differ despite a highly similar amino acid sequence (48). Additionally, TRP120 also contains Notch and Hedgehog SLiMs, which may also influence gene expression due to the intricate crosstalk between the pathways (49, 50).

To further establish the direct mechanism of Hippo regulation during infection, we determined that TRP120-Wnt-SLiM sufficiently induces active Yap. Notably, *E. chaffeensis,* TRP120, and TRP120-Wnt-SLiM induced similar Yap activity in THP-1 cells and PHMs, which is important to note since primary cells have a limited lifespan and THP-1 cells are a more practical alternative for laboratory studies. Additionally, we further defined the previously reported Wnt-SLiM (7), shortening the SLiM to 6 aa (from 17 aa) using BLAST analysis to detect a short region of homology among TRP120 and Wnt5a/3a ligands. Additionally, we determined that the histidine residue is essential for Yap and β-catenin activation. Various studies have indicated the importance of histidine in protein-protein interactions. In fact, histidine is known as the most active and versatile amino acid, is often the key residue in enzyme catalytic reactions, and is essential for protein interactions (51).

The shorter TRP120-Wnt-SLiM highlights shared amino acids between Wnt5a/3a that may be critical in binding Fzd receptors and activating signaling. In our previous study, we defined the TRP120-Wnt-SLiM based on sequence and functional similarities between TRP120 and Wnt8, since Wnt8 activates β-catenin and structural studies have defined many residues for Wnt8-Fzd binding (7, 52). However, many of the Wnt residues necessary for binding Fzd receptors are not conserved among the Wnt ligands (53). Additionally, Wnt5a and Wnt3a residues for Fzd binding are not well defined; however, these ligands are relevant to this study since they activate Yap (12). Identification of a SLiM with the capability to affect multiple pathways is new to science and will have a significant impact on how these ligand–receptor interactions are viewed by cell biologists and others.

In our investigation, TRP120-Wnt-SLiM exhibited stronger upregulation of Hippo gene targets than TRP120. This is likely due to higher molar concentrations of the SLiM sequence present in the TRP120-Wnt-SLiM treatment. To further support our results, we used Wnt-SLiM (QDVASH) to target Wnt signaling and determined that it does activate both Hippo and Wnt signaling, consistent with known Hippo-Wnt receptor overlap and crosstalk (20). Additionally, we used an anti-SLiM antibody that blocked Yap activation during *E. chaffeensis* infection and TRP120-Wnt-SLiM treatment, demonstrating the importance of the SLiM in Hippo regulation during infection and confirming that TRP120-Wnt-SLiM is the only SLiM utilized by *E. chaffeensis* to activate Yap.

In recent years, our laboratory has determined that TRP120 contains multiple SLiMs within the intrinsically disordered TRD that act as ligand mimics to regulate Wnt, Notch, Hedgehog, and Hippo signaling. *E. chaffeensis* likely contains multiple pathways activating SLiMs due to the intricate crosstalk between the pathways and the role each plays in regulating apoptosis to promote infection (20, 50, 54). SLiMs are disordered, short, linear sequences that contain a limited number of specificity-determining residues (55). Few mutations are necessary for the generation of new SLiMs, allowing rapid convergent evolution of SLiMs within proteins *de novo*, enabling rapid functional flexibility (56, 57). *E. chaffeensis* has likely convergently evolved TRP120 SLiMs to engage multiple cellular signaling pathways for redundancy and to influence anti-apoptotic signaling through different pathways. All defined TRP120 SLiMs activate conserved signaling pathways known to prevent apoptosis, which may be a strategy executed by *E. chaffeensis* to ensure host cell survival and productive infection.

TRP120 is a Wnt ligand mimic and directly interacts with the Fzd5 receptor (7). Wnt5a and Wnt3a ligands interact with the Fzd5 receptor, which can lead to the activation of Hippo and Wnt transcriptional regulators Yap and β-catenin, respectively. Further, while only Fzd-1, -2, and -5 are associated with Yap activation, most Fzd receptors are known to activate β-catenin (7, 12, 42, 43). Additionally, the co-expression of Fzd5 with the co-receptor tyrosine kinase ROR1 potentiates Fzd5 receptor-induced Yap activation (12). Previously, we demonstrated that *E. chaffeensis* survival depends on ROR1, which may be due to its role in the co-activation of Yap (7, 12). To better understand why *E. chaffeensis* interacts with the Fzd5 receptor and how it relates to Yap activation, we utilized Fzd5 receptor KO to demonstrate that the Fzd5 receptor is essential for Yap activation during infection. We found that the activation of *E. chaffeensis* and TRP120-Wnt-SLiM Yap is solely dependent on the Fzd5 receptor. Yap activation has been associated with Fzd-1, -2, and -5 in HEK293 cells (12, 21); however, the fact that Yap activation induced by *E. chaffeensis* and TRP120-Wnt-SLiM depends solely on the Fzd5 receptor may be related to fundamental differences in cell types (innate immune phagocyte vs. epithelial kidney cell). In contrast to our finding that Hippo relies completely on the Fzd5 receptor for signaling, β-catenin activation was only significantly reduced (~50%) in the Fzd5 receptor KO cells. This is consistent with reports demonstrating that multiple Wnt ligands and Fzd receptors are involved in β-catenin activation. Similarly, we have observed interactions between TRP120 and other Fzd receptors known to activate β-catenin (7).

Cellular apoptosis plays an important role as an innate defense mechanism against microbial infection. During infection, cells utilize apoptotic mechanisms for processing infected apoptotic bodies containing pathogens to facilitate antigen presentation and protective immunity (58). Preventing apoptosis is critical for obligate intracellular bacteria since maintaining a replicative niche is essential to complete the infection cycle. Obligate intracellular pathogens, including *Rickettsia, Anaplasma, Mycobacterium, Chlamydia,* and others, have evolved multiple regulatory mechanisms to inhibit host cell apoptosis, including regulation of mitochondria-mediated intrinsic apoptosis (58 - 65). Additionally, intracellular bacteria regulate the BCL-2 family of proteins to stabilize mitochondria and promote host cell survival. Recently, we demonstrated that *E. chaffeensis* activates the Hedgehog pathway to regulate mitochondria-mediated intrinsic apoptosis via BCL-2 and extend the host cell lifespan (10). *Chlamydia trachomatis* upregulates MCL-1 to inhibit Bax-induced apoptosis (66), and *M. tuberculosis* upregulates BCL-2 in macrophages during infection to prevent apoptosis (67).

Recently, investigations have demonstrated a major role for Hippo signaling in glucose metabolism to preserve mitochondrial stabilization and prevent apoptosis. To prevent apoptosis, the cell deactivates Hippo signaling to activate the transcriptional co-activator Yap to upregulate Hippo gene targets, including *SLC2A1*, which encodes the glucose transporter GLUT1. The upregulation of GLUT1 promotes glucose metabolism, which subsequently promotes the upregulation of BCL-xL (23, 26 - 28). Previous studies demonstrate that a reduction in GLUT1 protein expression increases Bax, Bak, Bim, and Bid (pro-apoptotic) and inhibits MCL-1 and BCL-xL (33). Additionally, *E. chaffeensis* infection and TRP120-Wnt-SLiM treatment increased GLUT1 and BCL-xL and decreased Bax levels. Further, we show that a small-molecule Yap inhibitor prevents *E. chaffeensis* from regulating GLUT1, BCL-xL, and Bax and induces a pro-apoptotic profile. These results reveal a novel anti-apoptotic mechanism by which *E. chaffeensis* modulates the Hippo pathway for infection by extending the host cell lifespan using glucose metabolism, which is consistent with the role of Hippo signaling in cell biology. Remarkably, *E. chaffeensis* regulates Hippo and Hedgehog to target various BCL-2 family proteins and inhibit intrinsic apoptosis, a remarkable redundancy resulting in comprehensive regulation of anti-apoptotic signaling for intracellular survival.

**Fig 12:**
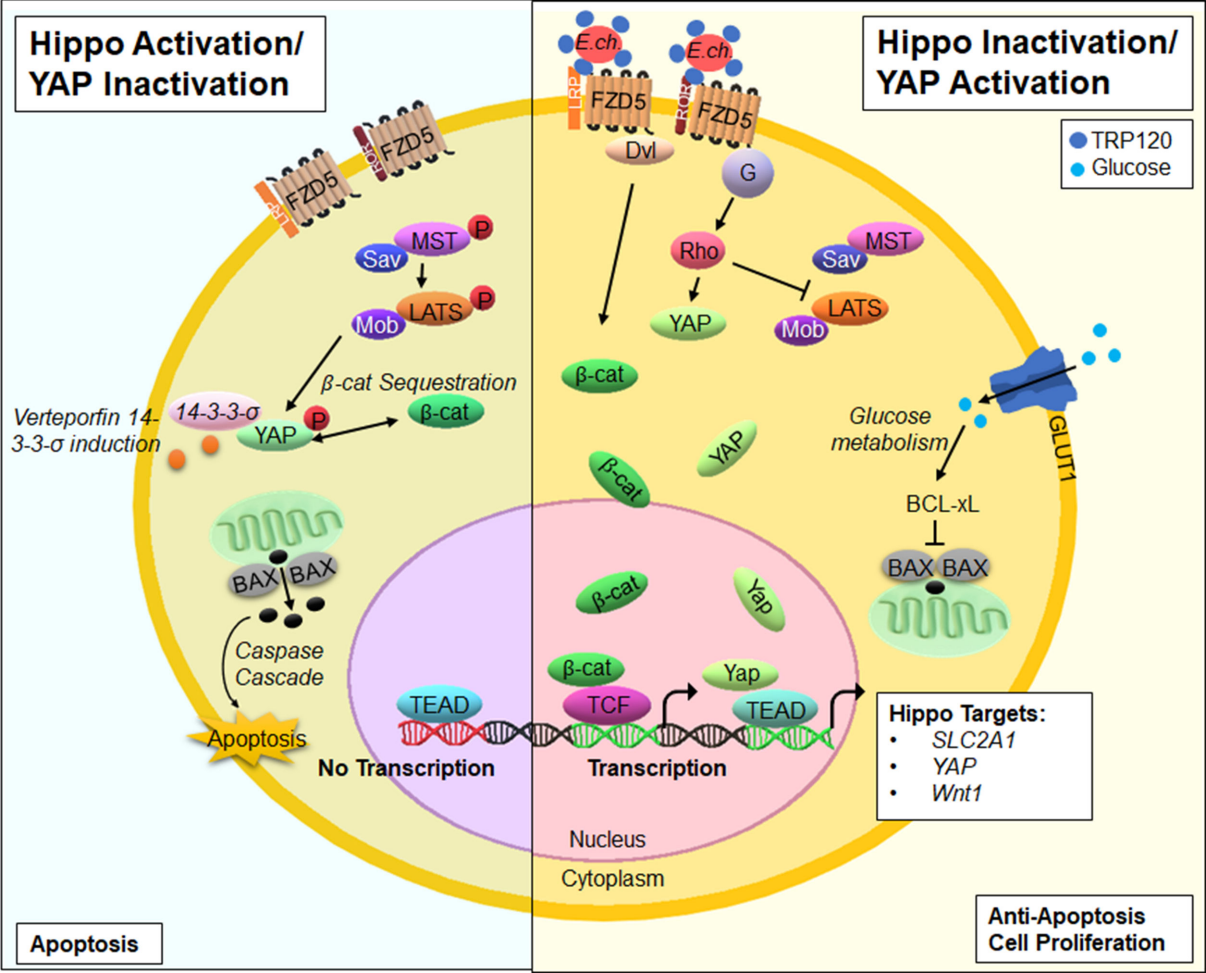
Model of Hippo activation/deactivation by *E. chaffeensis* TRP120 and the downstream effects on Yap and apoptosis. In normal cells, activation of Hippo signaling mediates cell fate through the phosphorylation and deactivation of Yap, which further leads to β-catenin deactivation and host cell apoptosis. In response, Wnt ligands bind the Fzd5 receptor at the cysteine-rich extracellular domain (ECD) to deactivate Hippo signaling, resulting in Yap and β-catenin nuclear translocation. During *E. chaffeensis* infection, surface-expressed TRP120 directly engages the Fzd5 receptor at the extracellular conserved cysteine-rich domain through a Wnt-SLiM repeated in the TRP120 TRD, thus activating transcription co-activators Yap and β-catenin to translocate freely to the nucleus to bind DNA and upregulate Hippo gene targets. Further, Yap nuclear translocation leads to the upregulation of the target gene *SLC2A1* (encodes GLUT1), thus upregulating GLUT-1 levels to increase glucose metabolism and prevent host cell apoptosis via BCL-xL inhibition of Bax.

The current study reveals a model of molecular mimicry in which a single bacterial SLiM phenocopies endogenous ligands to regulate multiple conserved signaling pathways. The discovery of SLiMs that phenocopy endogenous ligands provides a valuable tool for various fields to study cell signaling and cancer biology. Here, we characterize a TRP120-Wnt-SLiM that utilizes Hippo-Wnt pathway crosstalk to engage the Yap-GLUT1-BCL-xL axis to promote an anti-apoptotic profile (Fig. 12). This study demonstrates the importance of Hippo signaling in preventing apoptosis for ehrlichial replication and provides a potential new target for therapeutic development. The potential to use *E. chaffeensis* as a model to define the role of SLiM ligand mimicry and an evolutionary conserved eukaryotic signaling pathway will lead to a broader understanding of intracellular pathogen biology and provide mechanistic targets for intervention.

## MATERIALS AND METHODS

### Cell culture and *E. chaffeensis* cultivation

Human monocytic leukemia cells (THP-1; ATCC TIB-202) or PHMs were propagated in RPMI 1640 with L-glutamine and 25 mM HEPES buffer (Invitrogen, Carlsbad, CA), supplemented with 10% fetal bovine serum, and incubated at 37°C in a 5% CO_2_ atmosphere. Peripheral blood mononuclear cells were obtained from deidentified healthy human donors (Gulf Coast Regional Blood Center, Houston, TX), and PHMs were isolated using MACS negative selection (Miltenyi Biotec, Cambridge, MA). *E. chaffeensis* (Arkansas strain) was cultivated in THP-1 cells and PHMs as previously described (10).

### Protein sequence analysis

The NCBI Protein Basic Local Alignment Search Tool (Protein BLAST) was utilized for sequence alignment of TRP120 (NCBI accession number: AAO12927.1) and Wnt5a and Wnt3a amino acid sequences (NCBI accession numbers: AAH74783 and EAW69829).

### Recombinant proteins and peptides

*E. chaffeensis* recombinant full-length TRP120 (rTRP120-FL), TRP120 TRD (rTRP120-TR), or thioredoxin (rTrx; ctrl) were expressed in *Escherichia coli* and purified as described previously (9). rWnt5a (R&D Systems, Minneapolis, MN) and peptides (GenScript, Piscataway, NJ) were obtained from a commercial source. Synthesized peptides include TRP120-TR-Wnt5a (IKDLQDVASHESGVSDQPA; represents the entire homologous Wnt5a sequence), TRP120-TR (−) (SHQGETEKESGITESHQKEDEI; neg ctrl), TRP120-Wnt-SLiM (QDVASH), TRP120-Wnt-SLiM-mut (IKDLGAGAGAESGVS; Gly/Ala substitutions in the Wnt SLiM motif), and TRP120-Wnt-QDVAS (QDVAS).

### Antibodies and inhibitors

Antibodies used in this study include α-disulfide bond formation protein (Dsb) (68), α-TRP120-Wnt-SLiM (targets TRP120 sequence DLQDVASHESGVSDQPAQV) (7), α-TRP120 (7), α-active Yap (Abcam, Cambridge, UK), α-Yap, α-TEAD (1, 3, and 4) (Santa Cruz Biotechnology, Dallas, TX), α-active β-catenin, α-Fzd5 receptor, α-BCL-xL, α-Bax, α-Caspase-3, and -9 (Cell Signaling, Danvers MA), α-GLUT1 (Abcam, Cambridge, UK), α-GAPDH (MilliporeSigma, Burlington, MA), α-rabbit IgG (H + L) Alexa Fluor Plus 594, and α-mouse IgG (H + L) Alexa Fluor Plus 488 (Invitrogen Carlsbad, CA). Inhibition of the Hippo pathway was performed using Verteporfin (Thermo Fisher Scientific, Waltham, MA).

### Neutralization assay

*E. chaffeensis* or TRP120-Wnt-SLiM were incubated for 1 h or overnight, respectively, with 1.5 µg/mL of either α-TRP120-Wnt-SLiM antibody (targets TRP120 sequence SKVEQEETNPEVLIKDLQDVAS) or α-TRP120-PIS antibody (control), and then THP-1 cells were subsequently treated with each mixture for 10 h.

### RNAi and *Ehrlichia* quantification

All siRNAs were ON-TARGETplus SMARTpool (Dharmacon, Lafayette, CO). siRNA KD was performed as previously described (7, 10). Scrambled RNAi was used as an siRNA control. THP-1 cells were infected with cell-free *E. chaffeensis* (MOI 100) 24 h post-transfection. Cells were harvested at 24 hpi, and the ehrlichial load was determined using qPCR. All knockdowns were performed with three biological and technical replicates, and significance was determined using *t*-test analysis.

### Confocal microscopy

*E. chaffeensis-*infected (MOI 100) and uninfected THP-1 cells were seeded in T-150 flasks (Corning, Lowell, MA) and collected at 0, 4, 10, 24, and 48 hpi. THP-1 cells were treated with rTRP120-FL, rTrx (-), rWnt5a, TRP120-TR-Wnt5a, TRP120-TR (-), TRP120-Wnt-SLiM, or TRP120-Wnt-SLiM-mut (1 µg/mL) and collected 6 hpt for confocal microscopy as previously described (10). *E. chaffeensis-*infected, uninfected, rTRP120-FL, rTrx (-), rWnt5a, and TRP120-Wnt-SLiM and TRP120-Wnt-SLiM-mut peptide-treated PHMs were seeded in 12-well plates (Corning) containing a coverslip and incubated for 10 h. Cells were prepared for confocal microscopy as previously described (10) and stained with mouse anti-active Yap monoclonal antibody (1:200), rabbit anti-active β-catenin monoclonal antibody (1:100), and rabbit anti-Dsb antibody (1:500). Secondary antibodies were α-rabbit IgG (H + L) Alexa Fluor Plus 594 and α-mouse or rabbit IgG (H + L) Alexa Fluor Plus 488 (1:200). A Zeiss LSM 880 laser microscope was utilized to obtain all confocal laser micrographs and analyzed using Zen Black and Fiji software. Randomized areas/slides (*n* = 10) were used to detect active Yap. Experiments were performed with three biological and technical replicates.

### RNA isolation and cDNA synthesis

*E. chaffeensis*-infected (MOI 100), uninfected, rTRP120-FL, rTrx (-), rWnt5a, and TRP120-Wnt-SLiM (10 ng/mL or 1 µg/mL) cells were harvested at 24 h. Uninfected/untreated or rTrx (-)-treated cells were used as controls for infection and protein/peptide treatments. RNA isolation and cDNA synthesis were performed as previously described (10). Data were generated from three biological and technical replicates.

### Human Hippo signaling pathway PCR array

The human Hippo signaling target PCR array (Qiagen) was used to determine the expression of 84 key Hippo target genes. PCR arrays were performed according to the manufacturer’s protocol (Qiagen). Real-time PCR was performed using the RT^2^ Profiler PCR array and SYBR green master mix (Qiagen) using the QuantStudio 6 Flex Real-Time PCR system (Thermo Fisher Scientific). PCR data analysis was performed as previously described (7).

### Western immunoblot

Briefly, THP-1 cells were harvested, and lysates were prepared using CytoBuster protein extraction reagent (Novagen/EMD, Gibbstown, NJ) supplemented with a complete mini EDTA-free protease inhibitor (Roche, Basel, Switzerland) and phenylmethene-sulfonylfluoride PMSF (10 mM) (Sigma-Aldrich). Cell lysate protein concentrations were determined, and Western blots were performed as previously described (10) using α-Yap, α-TEAD (1, 3, and 4), α-Fzd5 receptor, α-BCL-xL, α-Bax antibodies (1:200), α-Caspase -3 and -9 antibodies (1:100), and α-GAPDH (1:10,000). Experiments were performed with three biological and technical replicates, and significance was determined by *t*-test analysis. All blots are presented in their original form but may have a loss of resolution due to image compression from the dock system, Chemidoc-it2 Imager.

### Fzd5 receptor knockout cells

CRISPR/Cas9 Fzd5 receptor KO THP-1 cells were obtained from a commercial source (Synthego, Redwood City, CA) and serially diluted to isolate a clonal population. Normal and Fzd5 receptor KO THP-1 cells were infected with *E. chaffeensis* (MOI 100) or treated with rWnt5a, TRP120-Wnt-SLiM, or TRP120-Wnt-SLiM-mut (1 µg/mL) and collected at 6 h for confocal and Western blot analysis.

### Real-time qPCR

The analysis of *SLC2A1* gene expression during infection was determined using real-time qPCR. THP-1 cells were infected with *E. chaffeensis* (MOI 100). Cells were harvested at 0, 3, and 24 hpi to examine gene expression during the entry and early replication phases. The fold change in *SLC2A1* from 0 to 3 or 24 hpi was calculated using the 2^−ΔΔ*CT*^ method and *C_T_* values for host *SLC2A1* and *GAPDH* genes as previously described (69).

### Hippo inhibitor infection analysis

*E. chaffeensis*-infected (MOI 50), uninfected, TRP120-Wnt-SLiM- and TRP120-Wnt-SLiM-mut-treated THP-1 cells were incubated with DMSO or Verteporfin (7 µg/mL) for 24 h, then cells were harvested for Western blot and Diff-Quik staining (Thermo Fisher Scientific). Diff-Quik images are in their original form, taken with a DP25 camera using cellSens. However, this software can create artifacts. Ehrlichial load was determined using qPCR as described above. Cell counts and viability were determined by the Cellometer Mini Brightfield Automated Cell Counter (Nexcelom, Lawrence, MA).

## SUPPLEMENTAL MATERIAL

Fig. S1 legend iai.00085-23-s0001.docx Full legend of Fig. S1.

Fig. S2 legend iai.00085-23-s0002.docx Full legend of Fig. S2.

Fig. S1 iai.00085-23-s0003.tif TRP120-Wnt-SLiM engages the Fzd5 receptor to activate Wnt signaling.

Fig. S2 iai.00085-23-s0004.tif TRP120 Wnt SLiM deletion mutant does not activate Yap or β-catenin.

ASM does not own the copyrights to Supplemental Material that may be linked to, or accessed through, an article. The authors have granted ASM a non-exclusive, world-wide license to publish the Supplemental Material files. Please contact the corresponding author directly for reuse.
