# Supplementary material for: Ehrlichia Wnt SLiM ligand mimic deactivates the Hippo pathway to engage the anti-apoptotic Yap-GLUT1-BCL-xL axis
Source: Infect Immun. 2023 Aug 2;91(9):e00085-23. doi: 10.1128/iai.00085-23 (PMC10501218; doi:10.1128/iai.00085-23)
Supplement: Fig. S1 legend — Full legend of Fig. S1. [file iai.00085-23-s0001.docx]

**Fig. S1. TRP120 Wnt SLiM engages the Fzd5 receptor to activate Wnt signaling.**

(A-B) Confocal immunofluorescence microscopy of *E. chaffeensis*-infected (MOI 100) or SLiM treated (1 μg/mL) THP-1 cells compared to untreated (-) and Wnt5a-treated (+) THP-1 cells stained with active β-catenin antibody. (A) The micrograph shows increased levels of active β-catenin (green) in Wnt5a (+), infected, and TRP120-Wnt-SLiM-treated, but not in TRP120-Wnt-SLiM-mut-treated THP-1 cells (6 hpt)(scale bar = 10 μm). (B) Confocal immunofluorescence microscopy of the Fzd5 receptor knockout (KO) THP-1 Fzd5 receptor KO cells were harvested (6 hpt) and immunostained with active β-catenin antibody (green). (A-B) Experiments were performed with three biological and technical replicates. Randomized areas/slide (n=10) were used to detect active β-catenin nuclear translocation. (C) Intensity graphs demonstrate the mean nuclear accumulation of active β-catenin in respective THP-1 cells. THP-1 FZD5 receptor KO cells have significantly less β-catenin. Analysis was performed using ImageJ and determining mean grey value from randomized areas/slide (n=10). Data are represented as means ± SD (****p*< 0.001).
