# Supplementary material for: Ehrlichia Wnt SLiM ligand mimic deactivates the Hippo pathway to engage the anti-apoptotic Yap-GLUT1-BCL-xL axis
Source: Infect Immun. 2023 Aug 2;91(9):e00085-23. doi: 10.1128/iai.00085-23 (PMC10501218; doi:10.1128/iai.00085-23)
Supplement: Fig. S2 legend — Full legend of Fig. S2. [file iai.00085-23-s0002.docx]

**Fig. S2. TRP120 Wnt SLiM deletion mutant does not activate Yap or β-catenin**

(A-B) Confocal immunofluorescence microscopy of TRP120-Wnt-SLiM His deletion mutant (TRP120-Wnt-QDVAS) peptide-treated (1 μg/mL) THP-1 cells compared to untreated (-) and Wnt5a-treated (+) THP-1 cells and stained with active Yap or β-catenin antibody. The micrograph shows no significant change in active Yap or β-catenin levels in TRP120-Wnt-SLiM His deletion mutant-treated cells compared to untreated (-) THP-1 cells (6 hpt)(scale bar = 10 μm). Experiments were performed with three biological and technical replicates. Randomized areas/slide (n=10) were used to detect active Yap or β-catenin nuclear translocation. (C) Intensity graphs demonstrate the mean nuclear accumulation of active Yap or β-catenin in respective THP-1 cells. Analysis was performed using ImageJ and determining mean grey value from randomized areas/slide (n=10). Data are represented as means ± SD (****p*< 0.001).
