# Supplementary figures and images for: Ehrlichia Wnt SLiM ligand mimic deactivates the Hippo pathway to engage the anti-apoptotic Yap-GLUT1-BCL-xL axis
Source: Infect Immun. 2023 Aug 2;91(9):e00085-23. doi: 10.1128/iai.00085-23 (PMC10501218; doi:10.1128/iai.00085-23)

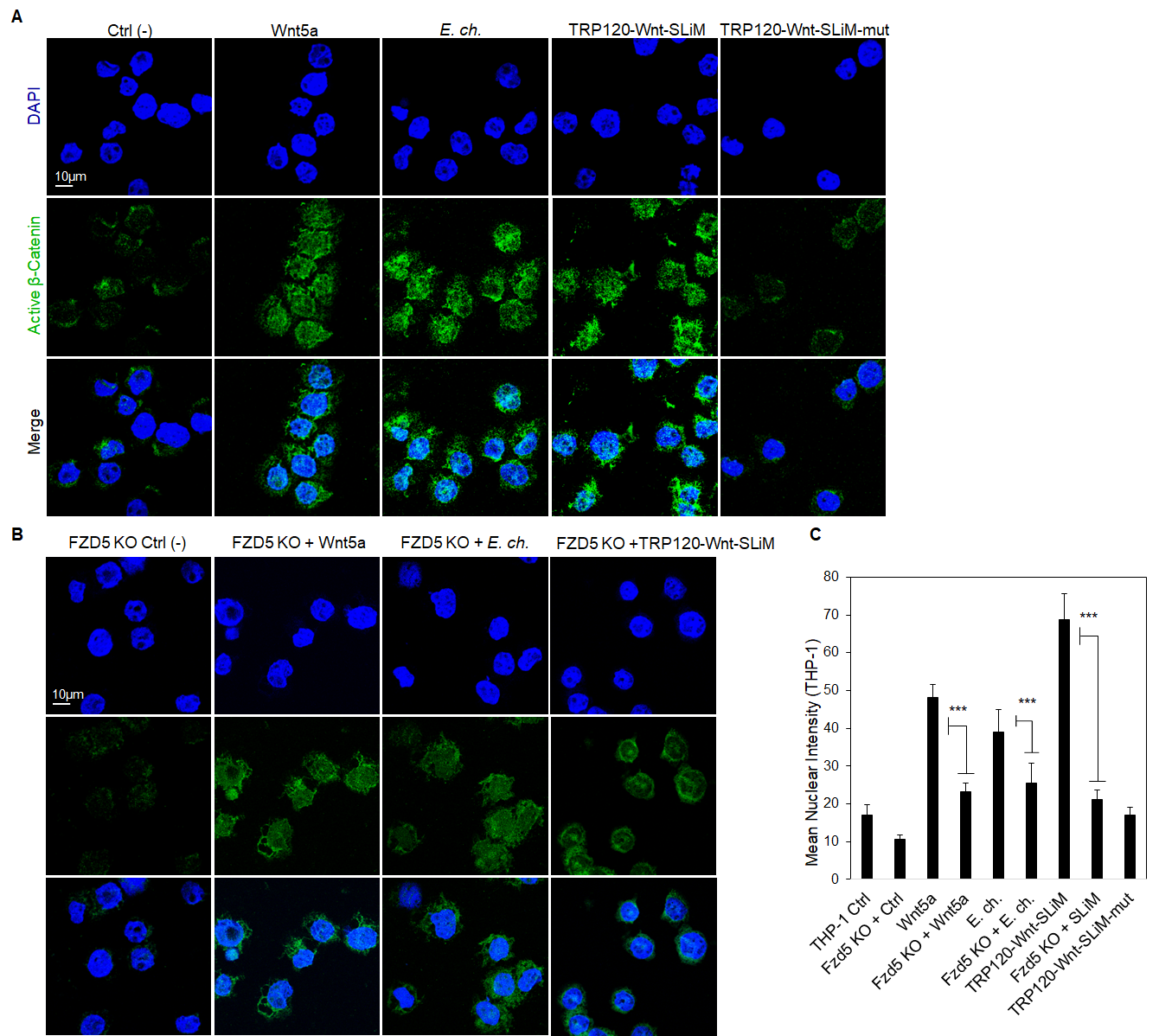

Supplement: Fig. S1 — TRP120-Wnt-SLiM engages the Fzd5 receptor to activate Wnt signaling. [file iai.00085-23-s0003.tif]

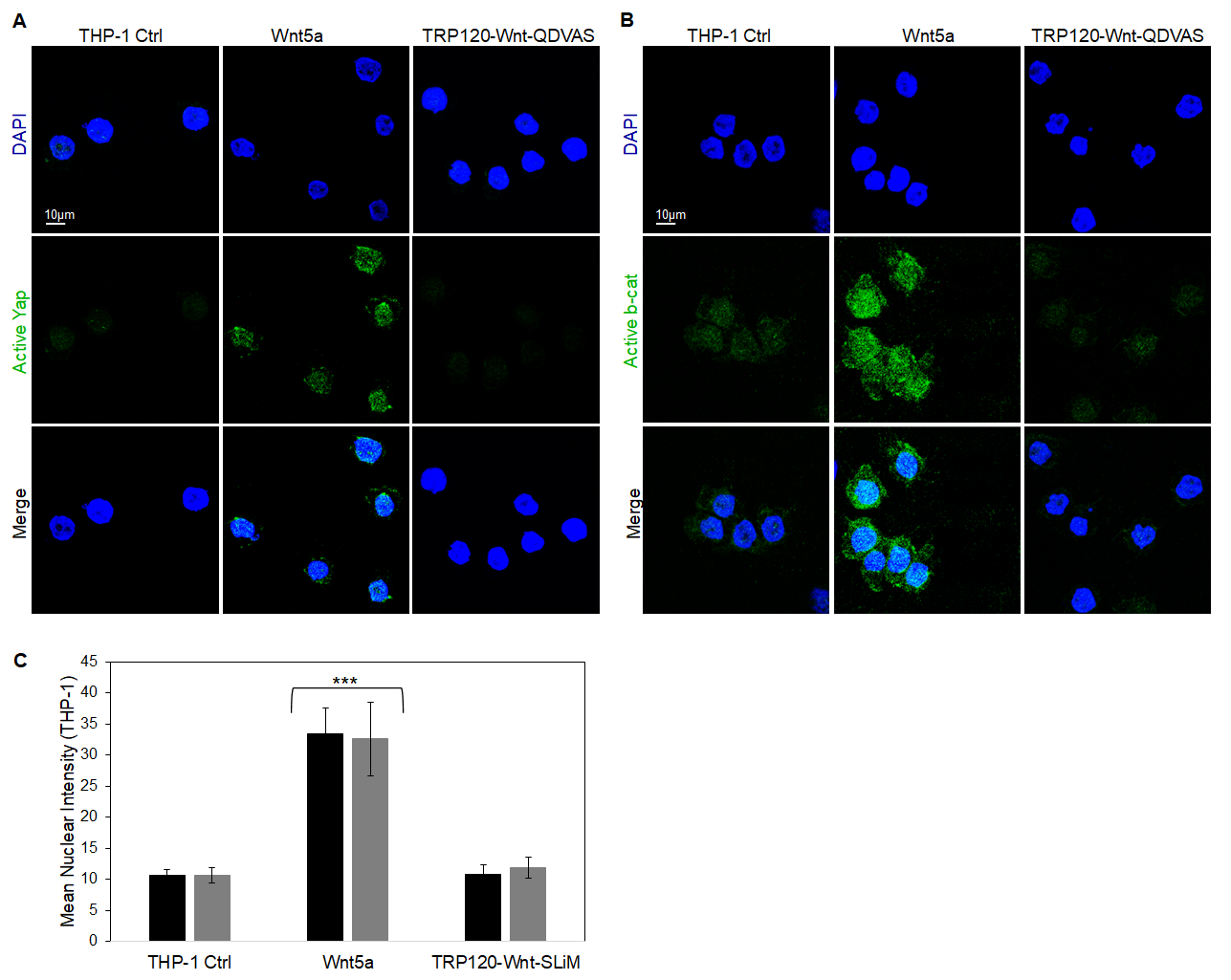

Supplement: Fig. S2 — TRP120 Wnt SLiM deletion mutant does not activate Yap or β-catenin. [file iai.00085-23-s0004.tif]
